# Latent Trajectories of Positive, Negative Symptoms and Functioning in Early Intervention Services for First-Episode Psychosis: A 2-Year Follow-Up Study

**DOI:** 10.1093/schbul/sbaf045

**Published:** 2025-05-04

**Authors:** Olivier Percie du Sert, Joshua Unrau, Manish Dama, Lena Palaniyappan, Jai Shah, Ridha Joober, Delphine Raucher-Chéné, Ashok Malla, Martin Lepage

**Affiliations:** Prevention and Early Intervention Program for Psychoses, Douglas Research Centre, Montreal, Quebec, H4H 1R3, Canada; Department of Psychiatry, McGill University, Montreal, Quebec, H3A 0G4, Canada; Prevention and Early Intervention Program for Psychoses, Douglas Research Centre, Montreal, Quebec, H4H 1R3, Canada; Prevention and Early Intervention Program for Psychoses, Douglas Research Centre, Montreal, Quebec, H4H 1R3, Canada; Prevention and Early Intervention Program for Psychoses, Douglas Research Centre, Montreal, Quebec, H4H 1R3, Canada; Department of Psychiatry, McGill University, Montreal, Quebec, H3A 0G4, Canada; Prevention and Early Intervention Program for Psychoses, Douglas Research Centre, Montreal, Quebec, H4H 1R3, Canada; Department of Psychiatry, McGill University, Montreal, Quebec, H3A 0G4, Canada; Prevention and Early Intervention Program for Psychoses, Douglas Research Centre, Montreal, Quebec, H4H 1R3, Canada; Department of Psychiatry, McGill University, Montreal, Quebec, H3A 0G4, Canada; Prevention and Early Intervention Program for Psychoses, Douglas Research Centre, Montreal, Quebec, H4H 1R3, Canada; Department of Psychiatry, McGill University, Montreal, Quebec, H3A 0G4, Canada; Prevention and Early Intervention Program for Psychoses, Douglas Research Centre, Montreal, Quebec, H4H 1R3, Canada; Department of Psychiatry, McGill University, Montreal, Quebec, H3A 0G4, Canada; Prevention and Early Intervention Program for Psychoses, Douglas Research Centre, Montreal, Quebec, H4H 1R3, Canada; Department of Psychiatry, McGill University, Montreal, Quebec, H3A 0G4, Canada

**Keywords:** schizophrenia, remission, heterogeneity, data-driven, structural equation modeling, growth mixture modeling

## Abstract

**Background:**

From the first episode (FEP), the course of psychosis is marked by substantial heterogeneity of clinical and functional outcomes which poses significant challenges in providing prognostic guidance to patients and families. To better understand such heterogeneity within the context of early intervention services (EIS), this study aimed to examine latent trajectories of positive and negative symptoms and functioning among FEP individuals undergoing EIS.

**Study Design:**

The Prevention and Early Intervention Program for Psychoses (PEPP-Montreal) is a 2-year EIS for FEP that conducted longitudinal assessments of 689 individuals aged 14-35, including sociodemographics, cognition, psychopathology, and functioning. Latent growth mixture modeling was used to identify distinct patterns of clinical and functional trajectories. The inter-relationship between trajectories, and the association of trajectory membership with baseline characteristics and distal outcomes were investigated using the manual 3-step approach.

**Study Results:**

Two positive symptom trajectories (*Stable-low*—32%, *Fluctuating*—68%,), 3 negative symptom trajectories (*Decreasing*—41%, *Fluctuating*—15%, and *Stable-high*—44%), and 2 functioning trajectories (*Increasing*—57%, *Stable-moderate*—43%) were identified. Early treatment response, particularly on negative symptoms, consistently and strongly predicted better outcome trajectories (OR = [3.4-5.5]). Trajectories of higher symptom severity were associated with trajectory of worse functioning (RR = [1.5-2.2]), which exhibited lower rates of clinical and functional remission.

**Conclusion:**

These findings offer insights into clinically meaningful subgroups of individuals that could inform the prognosis of FEP and the development of individually tailored EIS. Individuals who do not show early improvement in negative symptoms may benefit from earlier psychosocial interventions specifically targeting actionable factors that contribute to secondary negative symptoms.

## Introduction

The first episode of psychosis (FEP) typically occurs during adolescence or early adulthood,^1^ derailing the normal trajectory of life as it often persists into adulthood and beyond.^2^ The subsequent 2-5 years are considered a “critical period” of neurobiological and psychosocial plasticity, providing a window of opportunity for intervention. During this period, timely and appropriate treatment may result in favorable and sustainable outcomes.^3^ Seminal research has suggested that early illness trajectories are the strongest predictor of outcomes over the following 15-20 years.^4,5^ Hence the importance of investigating trajectories of outcomes during the critical period, particularly within the context of early intervention services (EIS) for psychosis.

Early intervention services provide integrated and specialized care during the first 2 years following FEP, aiming to reduce clinical symptoms, improve functioning, and mitigate long-term disability.^6,7^ Since the inception of EIS, clinical remission rates have improved.^8–10^ However, while systematic and meta-analytic reviews have demonstrated the short-term superiority of EIS compared to regular care, the reported effect sizes across most outcomes remain modest.^8,11–14^ Despite 49%-59% of individuals achieving clinical remission,^9,15^ 50%-75% continue to grapple with lasting functional impairments.^16^ This not only highlights the potential for optimizing EIS but also underscores the substantial heterogeneity in clinical and functional outcomes observed in the early course of psychosis.^9,17^ Such heterogeneity implies that EIS may have markedly different effects on different individuals. One-size-fits-all approaches are unlikely to succeed, emphasizing the need for personalized treatment strategies.

In addition, this heterogeneity poses significant challenges for clinicians in planning treatment and providing prognostic guidance to patients and families. Recent meta-analyses have largely failed to identify malleable predictors of clinical remission and functional recovery,^8,18,19^ with the exception of duration of untreated psychosis and attrition.^9,15,20^ Despite insight into some prognostic factors, none have proven sensitive or specific enough to reliably estimate an individual’s chance of recovery.^21^ Approaches that do not account for the intrinsic heterogeneity of FEP are unlikely to yield accurate prognoses, underscoring the need for identifying individual characteristics and other factors associated with both favorable and unfavorable outcome trajectories. Early identification of at-risk individuals who may need more intensive and targeted interventions is crucial for optimizing the effectiveness of EIS.^22^ Recognizing those most likely to achieve better outcomes is key to ensuring efficient resource allocation.

Linear mixed-effects models have become the standard methodology for analyzing longitudinal data, yet they have resulted in a limited understanding of the heterogeneity of outcome trajectories.^23,24^ Alternatively, studies have often relied on arbitrarily defined clinical categorizations rather than objective, data-driven approaches.^25,26^ Heterogeneity can be more effectively addressed using longitudinal latent growth modeling (LGM). Latent growth modeling captures both within- and between-individual variability, enabling the modeling of outcome trajectories with distinct patterns of change over time and distinguishing underlying differences between more homogeneous subgroups.^27^ This data-driven approach may capture a broader range of patient experiences, trajectories, and outcomes, providing clinicians with valuable insights for making prognoses and facilitating the selection of individualized interventions. Habtewold et al. (2020) conducted a comprehensive review of data-driven studies examining trajectories for positive, negative, and cognitive symptoms in schizophrenia spectrum disorders. Longitudinal studies identified 2-5 trajectories of positive and negative symptoms, characterized by stability, deterioration, relapsing, and improvement.^28–32^ In addition, 3-4 trajectories of functioning were reported, with mostly stable levels of impairment ranging from preserved to severe.^28,33,34^ Trajectories were associated with factors including age, sex, ethnicity, age of onset, diagnosis, duration of untreated psychosis, duration of illness, premorbid adjustment, global functioning, quality of life, and cognitive performance.^17^ However, few studies involved FEP cohorts, and only 2 studies^28,33^ were conducted within the 2- to 5-year critical period of EIS delivery.

This study sought to parse the heterogeneity of a catchment area-based sample of individuals experiencing FEP and undergoing 2 years of EIS during the critical period. First, we identified latent trajectories of positive and negative symptoms, as well as functioning. Second, we examined the interrelationships between these trajectories and explored the prognostic value of trajectory membership in relation to baseline characteristics and distal outcomes.

## Methods

### Clinical Setting

The Prevention and Early Intervention Program for Psychoses (PEPP-Montreal) is a 2-year, high-fidelity EIS for FEP serving a catchment area population of 350 000 in southwest Montreal, Canada. Being the only EIS in its catchment area, PEPP-Montréal is likely to serve a nearly complete treatment incidence sample. Core EIS components of PEPP-Montreal included a program designed to reduce treatment delays and provide rapid access to a package of multimodal evidence-based interventions delivered through assertive case management (ratio 20:1). This package included pharmacological (e.g., minimum effective dose of second-generation antipsychotics) and psychosocial interventions (e.g., cognitive behavior therapy; psychoeducation, group and family interventions; and individual placement and support program).^35,36^ Fidelity to the EIS delivery model, as defined by Addington et al. (2013),^6,7^ was established through monitoring of case manager-to-patient ratios, minimum number of clinical contacts per month, and the availability of a range of psychosocial interventions.^37,38^ This study was approved by the Ethics Board of the Douglas Mental Health University Institute and informed consent was obtained from all study participants.

### Participants

Between 2003 and 2018, PEPP-Montreal provided EIS to 689 individuals aged 14-35 years old, with a DSM-IV diagnosis of first episode of a non-affective or affective psychosis, who initially had no or minimal exposure to antipsychotic medication (< 30 days). Exclusion criteria at the time of admission were organic or substance-induced psychosis, history of central nervous system disorder, acquired brain injury, inability to speak either English or French fluently, IQ below 70, and inability to provide informed consent.

### Assessment

A comprehensive longitudinal assessment conducted over a 2-year follow-up yielded a detailed characterization of participants on multiple dimensions including sociodemographic, cognition, psychopathology, and functioning. Participants were assessed by trained research assistants at baseline, 1, 2, 3, 6, 9, 12, 18, and 24 months of follow-up.^39^ Quality controls were routinely implemented, and assessments of inter-rater reliability (estimated by percent agreement using adjacent categories) consistently showed satisfying agreement [0.75-0.92].^39^

#### Baseline, Cross-Sectional Assessment

*Sociodemographic:* Information included age, sex, ethnicity, relationship status, and years of education. Individuals were considered “Not in employment, education or training” (NEET)^40^ at program entry if they were not employed (whether full- or part-time), not in school, nor a stay-at-home parent. Living situations were dichotomized between independent (i.e., living alone, with a spouse, children, or friends) and dependent living (i.e., living with parents, extended family, in group or nursing home, being hospitalized or homeless). Socioeconomic status was defined using the Hollingshead Four-Factor Index (SES).^41^ Premorbid academic and social functioning from childhood through early adolescence was assessed retrospectively with the Premorbid Adjustment Scale (PAS).^42^  *Topography of first episode:* Information regarding the age of onset, mode of onset (i.e., insidious vs. acute), and the duration of untreated psychosis (DUP) was obtained using the Circumstance of Onset and Relapse Schedule.^43^ Age of onset was defined as the date when individuals first experienced a threshold level of psychotic symptoms for a duration of at least 1 week. An insidious mode of onset was defined if the period between the onset of the prodromal phase and the onset of psychosis was > 30 days.^44,45^ DUP was defined as the time between the onset of threshold-level psychotic symptoms and the start of adequate pharmacological treatment (i.e., 30 consecutive days of antipsychotic medication). Diagnoses of psychotic and other comorbid disorders were determined after 3 months through the Structured Clinical Interview for DSM-IV and confirmed 1 year into treatment by 3 senior psychiatrists (A.M., R.J., and J.S.). *Cognition:* Cognitive performance was evaluated following clinical stabilization (i.e., 6-12 months into the program), across all 7 MATRICS^46^ domains of cognition (i.e., verbal memory, visual memory, working memory, visual attention, processing speed, executive functioning, and social cognition) with the computerized CogState Research Battery.^47^ Full IQ was assessed using the Wechsler Abbreviated Scale of Intelligence (WASI).^48^

#### Longitudinal Assessment

*Functional outcome:* Functioning was measured at baseline, 12, and 24 months with the Social and Occupational Functioning Assessment Scale (SOFAS).^49^  *Clinical outcomes:* Psychopathology was assessed at baseline, 1, 2, 3, 6, 9, 12, 18, and 24 months. The severity of positive and negative symptoms was rated with the Scale of Assessment for Positive (SAPS)^50^ and Negative Symptoms (SANS).^51^ In addition, the severity of anxiety, depression, and mania symptoms was assessed with the Hamilton Anxiety Scale (HAS),^52^ the Calgary Depression Scale (CDS),^53^ and the Young Mania Rating Scale (YMRS), respectively. Insight was rated with the Scale to assess Unawareness of Mental Disorder (SUMD).^54^ Substance use was recorded with the Chemical Use, Abuse, and Dependence Scale (CUAD).^55^ Finally, antipsychotic usage was recorded, and dosage was converted to chlorpromazine equivalent according to recommendations^56^ while medication adherence was evaluated through pill-counting, participant and clinician reports.^57^  *Response, remission, and recovery:* Early response was defined as a 50% reduction in positive, negative, or total positive and negative symptoms severity from baseline to month 3.^58^ Symptom remission was established at month 24 using the Remission in Schizophrenia Working Group (RSWG) criteria, characterized by minimal severity of positive and/or negative symptoms (i.e., SAPS or SANS scores ≤ 2 on all global items, excluding the attention subscale), sustained for at least 6 months.^59^ Clinical remission was defined as sustained remission of both positive and negative symptoms for at least 6 months. Functional remission was characterized at 24 months using a cut-off of score for good functioning (i.e., SOFAS > 60)^4^ persisting for over 1 year. Individuals were considered in recovery if they met the criteria for both clinical and functional remission.

### Statistical Analyses

Intent-to-treat analyses were conducted, and missing data were handled using maximum likelihood under the assumption that data were missing at random (FIML). To account for the effect of treatment discontinuation, sensitivity analyses were performed by further excluding participants with more than 30% missing data. All statistical analyses were performed using R^60^ and Mplus^61^ software. The MplusAutomation package for R^62^ was used to automate the iterative model-fitting procedure and the manual 3-step approach while analyses were performed in Mplus. In accordance with open-science principles, which are integral to our institution,^63^ and as a means for promoting transparency, standardization, and reproducibility, all the code used in this study have been made open source and are readily accessible at https://github.com/OlivierPDS/MplusLGM.

#### Trajectory Analyses

Longitudinal latent growth modeling (LGM) was used to parse the heterogeneity and investigate the distinct trajectories of functional and clinical outcomes over the 2-year follow-up at PEPP-Montréal. Individual trajectory membership was determined based on SOFAS scores (i.e., 3 time points), or the sums of SAPS and SANS global scores (i.e., 9 time points). With the growing popularity of data-driven approaches in recent years, debates over best practices, correct specification, and potential misuse of these methods have ensued.^27,64,65^ In the latest guidelines, Van Der Nest et al. (2020) outline a standard model specification and selection strategy to address the risk of overfitting, which can result in an excessive number of extracted latent subgroups. To the best of our knowledge, the application of these guidelines has yet to be observed.^27^ This involved fitting increasingly less constrained LGMs in a systematic fashion to determine the optimal and most parsimonious set of model parameters.

First, a single-class growth curve model was estimated to represent the sample mean trajectory of outcome over time, against which subsequent models were compared. Second, class enumeration was performed by fitting a series of group-based trajectory models (GBTM) with an increasing number of classes. No more than 6 classes were expected based on previous research findings.^17^ Third, the model with the optimal class structure was extended in a latent class growth analysis by allowing free estimation of the residual variance across time, classes, and both. Fourth, class-invariant and class-variant random effects were added stepwise in growth mixture models (GMM) by allowing the variance and covariance of the growth factors to be estimated and to vary across classes. To mitigate the risk of local maxima, each model was rerun with twice the number of random starts (starting with a set of 500 and 500/4) until the best log-likelihood value was replicated within and between 2 runs.

Model selection was determined according to the Bayesian information criterion (BIC), the scaled entropy (sE), the average posterior probabilities (APPA), and the adjusted Lo–Mendel–Rubin bootstrap likelihood ratio test (aLMR-LRT). A significant aLMR test indicates that a *K* class model has a significantly better fit than a *K*-1 class model. Lower BIC values suggest a more parsimonious model, higher entropy (> 0.5) values imply a greater classification certainty of individuals, while higher APPA (> 0.7) are indicative of a good fit. Aside from fit statistics, interpretability was also taken into consideration, and models with classes accounting for less than 5% of the sample were rejected. Finally, the order of polynomials (i.e., linear, quadratic, and cubic) for the best-fitting model was refined by dropping iteratively the highest non-significant polynomial term based on Wald tests. Given the required number of timepoints, the pre-set polynomial order was assumed to be quadratic (3 timepoints minimum) for functional trajectories and cubic (4 timepoints minimum) for clinical trajectories. While heterogeneity in the overall distribution of the data is expected, normality was only assessed within classes of the optimal model using the multivariate skewness and kurtosis test.^66^ If required, corrected distributions were used accordingly to accommodate excessive skewness or kurtosis such as provided in Mplus.^67^

Interrelationships between trajectories of different outcomes were examined by plotting the cross-distribution of individuals for each combination of 2 trajectories. Risk ratios were computed to estimate the likelihood of individuals from one trajectory belonging to another.

#### Covariates Analyses

The association of trajectory membership with baseline predictors and distal outcomes was investigated following the manual 3-step approach.^68,69^ First, an unconditional model with no covariates was estimated such as previously described (i.e., class enumeration, model selection, and refining of polynomial order). Second, the misclassification error rates estimated through the logit approach were extracted from the optimal model. Third, covariates were added to the optimal model one at a time, while maintaining the known class structure by using the fixed logit values. This step allowed for the estimation of the effects of covariates while taking into account class uncertainty rates^70^ and preventing any change in the class distribution when new model parameters were added. Covariates were identified based on their relevance and significance from the existing literature, influencing outcomes, and remission and recovery trajectories.^17,19,71,72^ A range of covariates were investigated including sociodemographic factors (age, sex, ethnicity, IQ, education, employment, housing, relationship situations, and SES), first episode characteristics (age and mode of onset, DUP, hospitalization, baseline, and SUD diagnoses), as well as cognitive domains (verbal, visual, and working memory, visual attention, processing speed, executive functioning, and social cognition). Functional (i.e., PAS and SOFAS) along with clinical factors (i.e., SAPS, SANS, HAS, CDS, YMRS, SUMD, medication adherence, CPZ equivalent dose) were also analyzed. In addition, response, remission, and recovery variables were examined. For each investigated trajectory, the outcome modeled, as well as variables derived from that outcome (e.g., response and remission), were excluded from the covariate analyses to mitigate endogeneity bias. Multinomial logistic regressions were performed to identify predictors of latent class membership. Odd ratios were computed for the “better” trajectories in reference to the “worse” trajectory, as the exponentiation of the logistic regression coefficients. Finally, Wald chi-square tests were used to test for mean and threshold equality between latent trajectories in relation to distal outcomes at the end of the 2-year follow-up period. Standardized mean differences were computed as the ratio of the difference in estimated mean between trajectories by the estimated standard deviation within the sample. Bonferroni corrections were used to account for multiple testing.

## Results

### Characteristics of the Sample

An overview of the sociodemographic, functional, and clinical characteristics of enrolled participants is presented in Table 1.

**Table 1.** Baseline Characteristics of PEPP-Montreal Cohort (*N* = 689)

Characteristic	Mean (SD)/*n* (%)
Age	23.7 (4.8)
IQ	97.1 (15.1)
SES	52.8 (15.4)
Education (years)	12.5 (2.9)
Sex	
* Female*	208 (30%)
* Male*	481 (70%)
Ethnicity	
* Not visible minority*	394 (62%)
* Visible minority*	243 (38%)
Employment	
* NEET*	417 (66%)
* Non-NEET*	213 (34%)
Housing	
* Dependent*	302 (46%)
* Independent*	356 (54%)
Relationship	
* Single*	604 (89%)
* Not single*	72 (11%)
Age of onset	22.7 (4.8)
DUP (weeks)	14.6 (109.7)
Diagnosis	
* Affective Psychosis*	180 (29%)
* SSD*	437 (71%)
SUD	147 (37%)
* No*	255 (63%)
* Yes*	147 (37%)
PAS	0.2 (0.1)
SOFAS	40.6 (13.0)
SAPS	11.4 (3.2)
SANS	9.9 (3.8)
HAS	9.3 (6.9)
CDS	5.1 (4.9)
YMRS	24.3 (11.5)
SUMD	2.8 (1.2)
CPZeq	186.7 (184.0)

Abbreviations: CDS: Calgary Depression Scale, CPZeq: Chlorpromazine equivalent, DUP: Duration of untreated psychosis, HAS: Hamilton Anxiety Scale, NEET: Not in employment, education or training, PAS: Premorbid Adjustment Scale, SANS: Scale for the Assessment of Negative Symptoms, SAPS: Scale for the Assessment of Positive Symptoms, SES: Socioeconomic status, SOFAS: Social Occupational Functioning Scale, SUD: Substance use disorder, SSD: Schizophrenia spectrum disorder, SUMD: Scale to Assess Awareness in Mental Disorder, YMRS: Young Mania Rating Scale.

### Trajectory Analyses

Table 2 presents a summary of the optimal models derived from longitudinal LGM analyses that have demonstrated superior performance based on the BIC, sE, APPA, and aLMR-LRT. The table includes the model parameter specifications along with their corresponding fit indices, class counts, and proportions based on estimated posterior probabilities. Further details regarding the model selection process and all models estimated can be found in Supplementary Tables S1, S2, and S3.

**Table 2.** Summary of best-fitting latent growth mixture models *Note:* aLMR: Adjusted Lo-Mendell-Rubin likelihood ratio test, BIC: Baeysian information criterion, GBTM: Group-based trajectory model, GMM: Growth mixture model, SANS: Scale for the assessment of negative symptoms, SAPS: Scale for the assessment of positive symptoms, SOFAS: Social occupational functioning scale.

Outcome	*N*	Latent classes	Model	Parameters	Polynomial	Random effect	Residuals	BIC	aLMR *P* val	Class 1	Class 2	Class 3	Entropy
SAPS	679	2	GMM (class-variant)	33	Cubic	Intercept and linear slope	Free across time & classes	22,418	<.001	460 (68%)	219 (32%)		0.81
SANS	679	3	GMM (class-invariant)	18	Cubic (1, 2); linear (3)	Intercept and linear slope	Free across classes	22,283	<.001	104 (15%)	277 (41%)	298 (44%)	0.43
SOFAS	650	2	GBTM	8	Quadratic	0	Fix across time & classes	11,082	<.001	280 (43%)	370 (57%)		0.51

#### Trajectories of Positive Symptoms Severity (SAPS)

**Figure 1.**
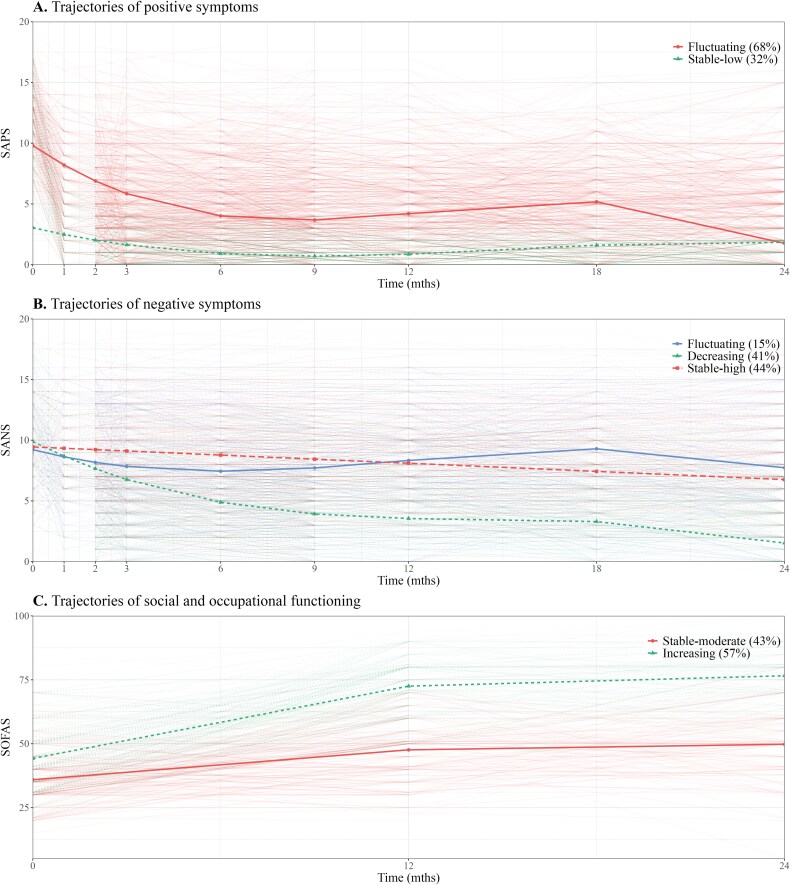
Estimated latent trajectories of outcomes over 2-year early intervention services*Note:* Observed individuals’ trajectories are plotted in the background.

 The most parsimonious model for characterizing trajectories of positive symptoms was a 2-class cubic GMM with class-variant random effects for the intercept and linear slope. The residual variance was free to vary across classes and over time (see Supplementary Table S1 for details). The *Stable-low* trajectory (SL: *n* = 219; 32%) began with low baseline symptom severity (m0 = 3.0), showed a progressive decrease within the first 6 months and remained stably low thereafter (m6-m24 = [0.6-1.1]). In contrast, the *Fluctuating* trajectory (F: *n* = 460; 68%) began with higher baseline symptom severity (m0 = 9.8), rapidly decreased within the first 6 months, then fluctuated to reach moderate severity (m24 = 4.2) by the end of the follow-up period. Positive symptom trajectories are illustrated in Figure 1A.

#### Trajectories of Negative Symptoms Severity (SANS)

Trajectories of negative symptoms were best modeled using a 3-class GMM with class-invariant random effects for the intercept and linear slope. The residual variance was relaxed across classes but not over time (see Supplementary Table S2 for details). Two trajectories exhibited a cubic growth pattern, while the effect of time was found to be linear in the third trajectory. Symptom severity started high (m0 = [9.2-9.8]) across all trajectories. The *Decreasing* trajectory (D: *n* = 277; 41%) showed steady improvement from baseline to 6 months, stabilizing thereafter, while the *Fluctuating* trajectory (F: *n* = 104; 15%) displayed successive periods of limited decrease and increase, both trajectories eventually reached a moderate level (m24 = [4.8-5.7]). The *Stable-high* trajectory (SH: *n* = 298; 44%) was characterized by a minimal decrease in symptom severity which remained stably high (m24 = 6.8) throughout the entire follow-up. Figure 1B provides an illustration of negative symptom trajectories.

#### Trajectories of Social and Occupational Functioning (SOFAS)

 A 2-class quadratic GBTM was found to best model functional trajectories. The residual variance was fixed across classes and over time (see Supplementary Table S3 for details). While both trajectories indicated major to serious functional impairment at baseline (i.e., m0 = [35.9-44.2]), both showed some improvement until reaching a plateau after the first year. However, the 2 trajectories differed in the rate of change over time, with the *Increasing* trajectory (I: *n* = 280; 43%) achieving twice as much improvement as the *Stable-moderate* trajectory (SM: *n* = 370; 57%). By the end of the follow-up, the *Increasing* trajectory indicated no more than a slight impairment (m24 = 76.4), whereas the *Stable-moderate* trajectory indicated serious to moderate impairment (m24 = 50). Functioning trajectories are represented in Figure 1C.

#### Cross-Distribution of Individuals between Latent Trajectories

**Figure 2.**
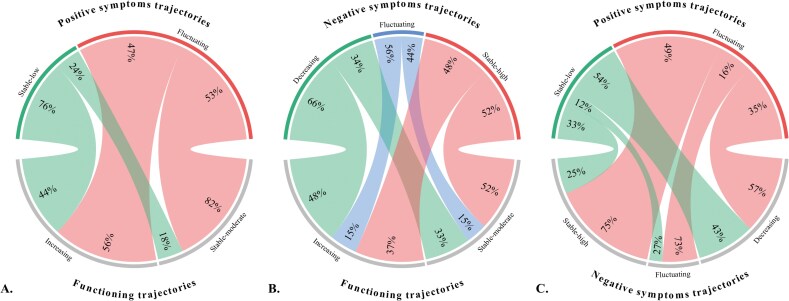
Cross-Distribution of Individuals between (A) Positive Symptoms and Functioning Trajectories, (B) Negative Symptoms and Functioning Trajectories, and (C) Positive and Negative Symptoms Trajectories *Note:* Each link represents the number of intersecting individuals between the two trajectories it connects, along with the corresponding proportions it represents in each of these trajectories. For example, 76% of individuals from the *Stable-low* trajectory of positive symptoms also belong to the *Increasing* trajectory of functioning, representing 44% of it.

The cross-distribution of individuals between trajectories of functioning, positive and negative symptoms are represented in Figure 2. Individuals with trajectories of higher symptom severity were at greater risk of following a trajectory of worse functioning. Those in the *Fluctuating* trajectory of positive symptoms were more than twice as likely to follow the *Stable-moderate* trajectory of functioning (RR = 2.23; 95%CI = [1.72-2.88]; *P* < .001), while the risk increased by 53% for those in the *Stable-high* trajectory of negative symptoms (RR = 1.53; 95%CI = [1.24-1.86]; *P* < .001). In contrast, trajectories with lower symptom severity were not as strongly associated with the trajectory of better functioning. Individuals in the *Stable-low* trajectory of positive symptoms had 61% higher chance to follow the *Increasing* trajectory of functioning (RR = 1.61; 95%CI = [1.42-1.82]; *P* < .001), while the chance only increased by 37% for individuals in the *Decreasing* trajectory of negative symptoms (RR = 1.37; 95%CI = [1.18-1.58]; *P* < .001).

### Covariates Analyses

#### Baseline Predictors of Class Membership

**Figure 3.**
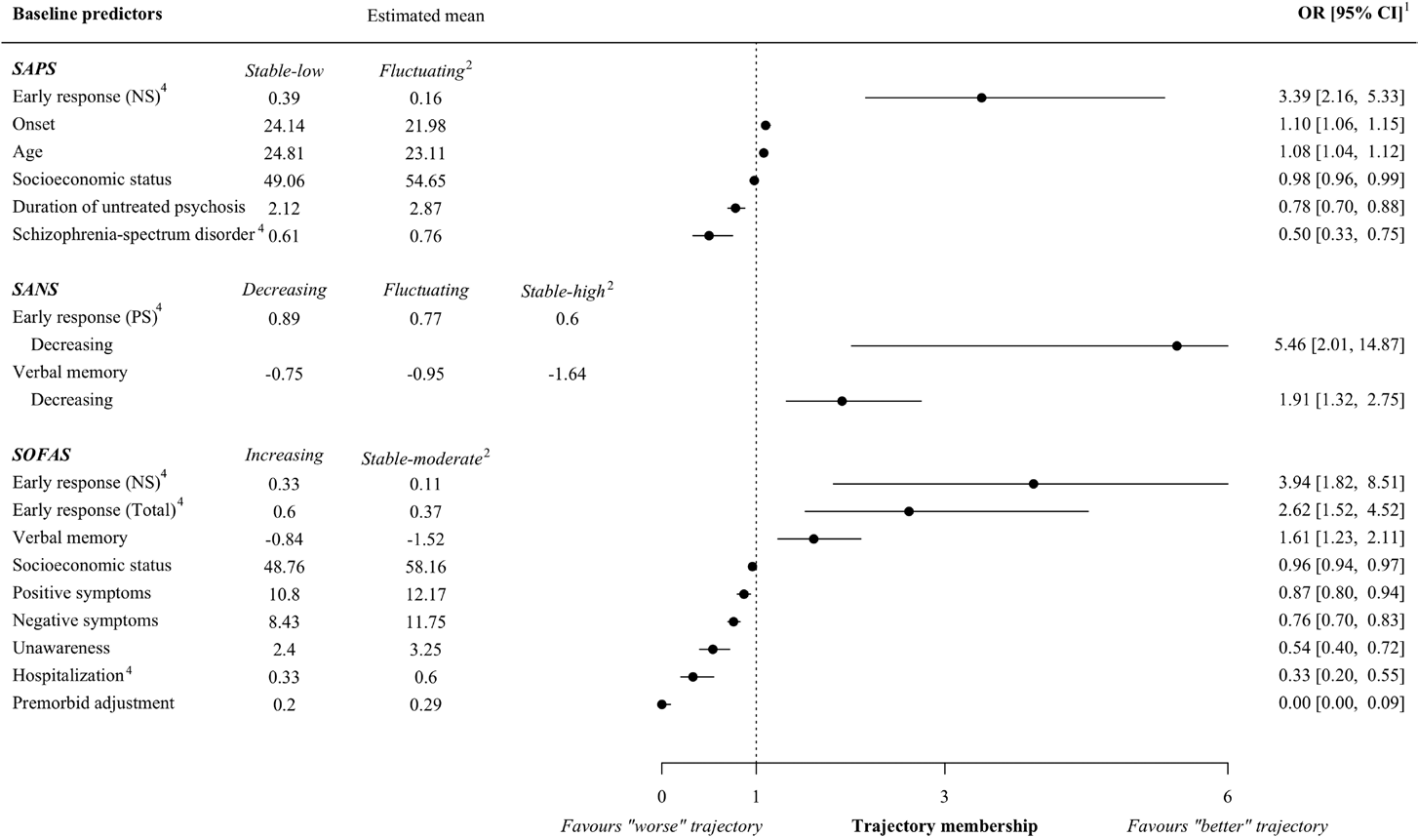
Baseline Predictors of Trajectory Membership *Note:* (1) All results are statistically significant using a Bonferroni-corrected threshold (*P* < .001). (2) Reference trajectory. (3) Log transformed. (4) Binary variable, proportion of cases coded as yes. CI: Confidence interval, OR: Odds ratio, PS: Positive symptoms, SANS: Scale for the Assessment of Negative Symptoms, SAPS: Scale for the Assessment of Positive Symptoms, SOFAS: Social Occupational Functioning Scale.

Results of the multinomial logistic regression analyses that survived Bonferroni corrections are reported in Figure 3. Observed means and non-significant results can be found in Supplementary Tables S4 and S5.

Early response to treatment emerged as the strongest predictor of belonging to better clinical trajectories. Individuals who experienced a 50% reduction of negative symptoms within the first 3 months were, respectively, 3 times more likely to have a *Stable-low* trajectory of positive symptoms. In addition, the odds of having a *Decreasing* trajectory of negative symptoms were increased by 5.5 for individuals who experienced early response to treatment of positive symptoms. In contrast, younger individuals diagnosed with SSD at an earlier age, with lower socioeconomic status and longer DUP, tended to exhibit a worse trajectory of positive symptoms. In addition, deficits in verbal memory were linked to worse trajectories of negative symptoms.

Better premorbid adjustment from childhood through early adolescence, along with baseline employment strongly predicted membership in the *Increasing* trajectory of functioning. Early response to treatment of negative symptoms and lower deficits in verbal memory also significantly influenced the likelihood of belonging to this trajectory. In contrast, individuals who were hospitalized at intake, with higher severity of positive and negative symptoms along with lower insight had an increased risk of displaying a worse trajectory of functioning.

#### Distal Outcomes at the End of Early Intervention Services

**Figure 4.**
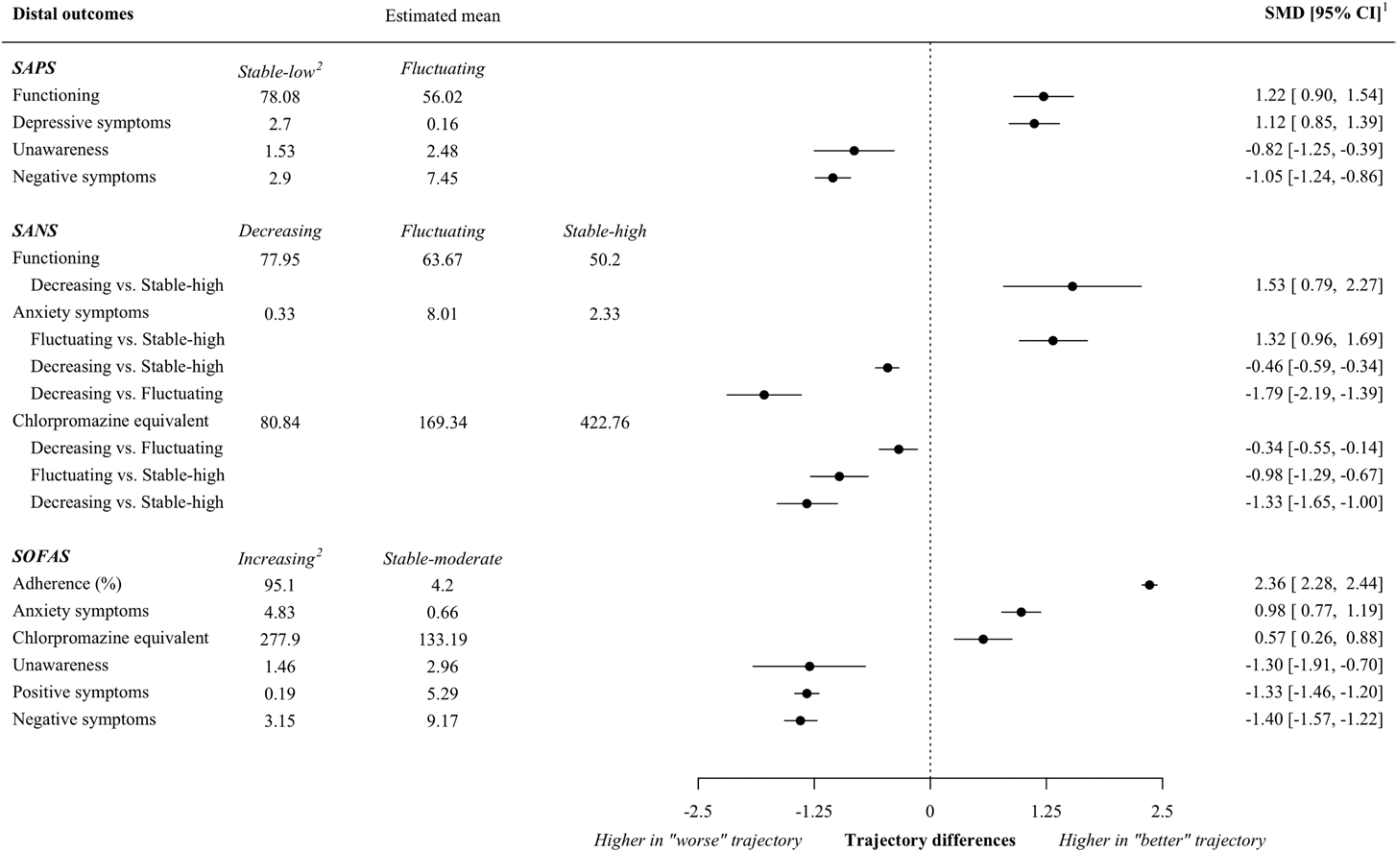
Trajectory Differences in Distal Outcomes at the End of the 2-Year Follow-Up Period *Note:* (1) All results are statistically significant using a Bonferroni-corrected threshold (*P* < .005). (2) Reference trajectory. CI: Confidence interval, SANS: Scale for the Assessment of Negative Symptoms, SAPS: Scale for the Assessment of Positive Symptoms, SOFAS: Social Occupational Functioning Scale, SMD: Standardized Mean Difference.

Standardized mean differences for continuous distal outcomes that survived Bonferroni corrections are reported in Figure 4. Observed means can be found in Supplementary Table S6.

By the end of the follow-up period, individuals in trajectories of lower severity of positive and negative symptoms not only demonstrated higher functioning but were also 15-20 times more likely to achieve functional remission respectively (OR = 15.75; CI = [7.88-31.50]; *P* < .001, OR = 20.03; CI = [3.42-117.10]; *P* < .001). In addition, individuals in the *Stable-low trajectory* of positive symptoms experienced lower negative symptoms and were 5 times more likely to achieve remission of negative symptoms (OR = 5.74; CI = [3.37-9.78]; *P* < .001).

Conversely, individuals following a trajectory of higher functioning experienced lower positive and negative symptoms by the end of the 2-year follow-up and had an extremely high likelihood of being in clinical remission (infinite OR; *P* < .001). They also showed better insight, were more adherent to medication, and were prescribed higher dosages of antipsychotics than individuals with a poorer trajectory of functioning.

#### Sensitivity Analyses

Following the exclusion of participants with more than 30% of missing data, the models selected, and the class distribution of identified trajectories remained identical to the main results (Supplementary Table S7).

## Discussion

This study examined latent trajectories of clinical and functional outcomes of individuals receiving EIS for FEP during the critical period. Despite substantial heterogeneity in individuals’ trajectories, results revealed discernible patterns of symptoms and functioning over the 2-year follow-up period. Two trajectories of positive symptoms, 3 trajectories of negative symptoms, and 2 trajectories of functioning were modeled through a systematic data-driven approach. Early treatment response consistently and strongly predicted membership in trajectories characterized by lower symptom severity. Conversely, belonging to trajectories of higher symptom severity significantly increased the risk of simultaneously following a trajectory of worse functioning. By the end of the follow-up period, individuals with a worse trajectory of functioning exhibited lower rates of both clinical and functional remission.

### Trajectories of Positive Symptoms

Two trajectories of positive symptoms were identified, accounting for individuals with either *Stable-low* (32%) or *Fluctuating* (68%) symptom severity. This finding aligns with the sole other study examining 2-year latent trajectories of positive symptoms during EIS for FEP, which identified 2 trajectories characterized by rapid and progressive decrease.^28^ Studies conducted outside EIS settings may have reported continuously high and unresponsive trajectories possibly due to the inclusion of chronic SSD patients treated with first-generation antipsychotics.^73–75^ While antipsychotic medication is the first-line treatment for positive symptoms, responses to treatment are heterogeneous.^22,76–81^ Higher response rates have been reported among drug-naïve^58^ and FEP patients,^82^ with treatment response typically observed within the first 2-4 months,^83,84^ consistent with our findings.

The *Stable-low* trajectory was associated with an early response to treatment of negative symptoms as well as higher functioning throughout the follow-up period. Although a reduction of positive symptoms can improve secondary negative symptoms,^85,86^ both trajectories initially decreased, suggesting a threshold-dependent rather than dose-dependent relationship. This may have clinical implications when assessing relapse risk for patients who often wish to stop medication following initial progress.^87^ Early improvements in secondary negative symptoms could serve as an actionable prognostic factor, indicating a clinically meaningful reduction of positive symptoms while predicting sustained clinical and functional remission.

The *Fluctuating* trajectory was associated with younger age, earlier onset, SSD diagnoses lower socioeconomic status and longer DUP, all of which are known risk factors of poor antipsychotic response,^58^ frequent relapses,^88^ and treatment resistance.^89,90^ Early identification of at-risk individuals may assist in the timely provision of appropriate management options. However, given reduced chances of success, clinicians should consider not only dose escalation, augmentation, or switching strategies,^91^ including the prescription of clozapine,^92,93^ but also psychosocial interventions such as cognitive behavior therapy for psychosis.^94–96^ These interventions should aim specifically at alleviating early on the distress and beliefs associated with positive symptoms that may be responsible for secondary negative symptoms.

### Trajectories of Negative Symptoms

Three trajectories of negative symptoms were identified, characterized by *Decreasing* (41%), *Fluctuating* (15%), and *Stable-high* (44%) symptom severity. While similar studies have also reported 3 trajectories,^31,74,97^ others have identified a fourth trajectory^28,30,32,73^ systematically accounting for a minority of individuals (i.e., < 5%). Notwithstanding potential model overfitting, the clinical significance of such a small subgroup is debatable. These results emphasize the importance of treating negative symptoms as heterogeneous and multi-dimensional rather than a unitary construct, broadly defined and unlikely to lead to the development of effective interventions.

The *Decreasing* trajectory was strongly associated with an early response to treatment on positive symptoms, as well as lower verbal memory deficits and higher functioning throughout the follow-up period. This is consistent with the improvement of secondary negative symptoms following treatment of positive symptoms. It also aligns with previous research showing that verbal memory is not only associated with negative symptoms^98,99^ but mediates the relationship between premorbid adjustment and functioning via the remission of positive symptoms.^98,100,101^ Patients with negative symptoms may benefit from cognitive remediation beyond cognitive improvements, by targeting specific domains of cognitive functioning, such as verbal memory.^102–104^

The *Fluctuating* and *Stable-high* trajectories exhibited persistent negative symptoms. Although these appear to be primary in 25% of individuals minimally affected by positive symptoms, they may be secondary and possibly stemming from unresolved positive symptoms for the remaining 75% (Figure 2C). While treatment options for negative symptoms are limited,^85,105^ these findings are encouraging as they highlight the unmet therapeutic potential of addressing secondary negative symptoms in a large proportion of individuals. Psychosocial interventions should target specifically actionable factors that cause or contribute to secondary negative symptoms, notably positive symptoms and cognitive deficits.

### Trajectories of Social and Occupational Functioning

Two trajectories were identified, characterized by *Increasing* (57%) and *Stable-moderate* (43%) functioning. Previous studies reported 3-4 trajectories showing distinct levels of improvements within the first year of treatment,^102^ followed by a plateauing course.^24,33,34,106,107^ Few studies reported a deteriorating trajectory, systematically accounting for less than 5% of the sample.^28,33,34^ While a ceiling effect of treatment may be at play, these findings underscore the importance of improving functioning early within a critical window of opportunity.

Early response of treatment on negative symptoms was strongly associated with the *Increasing* trajectory along with verbal memory and clinical remission. Given the pervasive impact of negative symptoms on social functioning,^71,108^ timely interventions to specifically address negative symptoms early during the provision of EIS are required.^109–111^ This is crucial because individuals who do not exhibit early improvement of negative symptoms not only face a worse trajectory of functioning, but their functioning also appears to be more strongly affected by positive symptoms, ultimately lowering their chances of remission. While clinical symptoms may moderate the association between neurocognition and functional outcomes,^112^ cognitive remediation has been shown to improve both negative symptoms and social functioning in FEP,^113^ especially when delivered as part of broader, multi-component EIS.^10^

Baseline predictors associated with the *Stable-moderate* trajectory included poor premorbid adjustment, hospitalization, lower insight, as well as higher positive and negative symptoms. These risk factors, along with the overall severity of psychopathology have been previously linked to poor functional outcomes.^71,72,114^ Evidence further supports that individuals with trajectories of higher symptom severity present a greater risk of impaired functioning throughout the follow-up. Our findings suggest that individuals with the most severe overall clinical presentation at admission require closer clinical attention as they are particularly at risk of experiencing persistent functional disability.

However, 39% and 55% of individuals in trajectories of lower positive and negative symptom severity still failed to reach functional remission, and 56% of individuals in the trajectory of better functioning did not achieve clinical remission (Supplementary Table S2). These findings confirm that, while clinical remission is closely related to functional remission, it is neither a necessary prerequisite nor sufficient on its own to achieve functional remission.^115^ A significant proportion of individuals demonstrate functioning that is not dependent on the severity of clinical symptoms, highlighting the contribution of factors beyond psychopathology. While this likely includes neurocognitive factors, social support, resilience, and coping strategies are also important in determining functional outcomes. PEPP-Montréal’s recovery-oriented and multidisciplinary approach provides a wide range of psychosocial interventions aimed at promoting individuals’ reintegration into community and social life.^36^ This may be instrumental in helping individuals reach functional milestones, regardless of symptom remission. This may fit into a broader process of personal recovery, wherein individuals find ways to cope with the distress of ongoing symptoms and overcome the challenges posed by psychosis to lead rewarding and fulfilling lives.^116^

### Limitations, Strengths, and Future Directions

Several limitations should be considered. First, there was a notable proportion of missing data in SOFAS (i.e., 34%), SAPS (i.e., 30%), and SANS (i.e., 30%) assessments. However, sensitivity analyses yielded results consistent with the primary analysis, indicating that the impact of attrition was minimal. Second, functional and clinical remission rates may have been overestimated because criteria were only verified at 12 and 24 months or 18 and 24 months, respectively, with no verification in-between. However, this approach is likely to be less overestimated than using no duration criteria of remission. Third, the sample included individuals with affective psychosis, which may have influenced the distribution across trajectories. However, latent trajectory analyses are specifically designed to account for such heterogeneity. Our findings indicate that diagnosis was only associated with positive symptom trajectories, highlighting its limited prognostic value for negative symptoms and functioning. Furthermore, this approach enabled the identification of transdiagnostic prognostic factors at program entry, a period before formal diagnoses can typically be established, especially considering the potential for diagnostic instability.^117^ This speaks to the ecological validity of our findings. A major strength of this observational study lies in the use of a large, well-characterized, catchment-area-based sample, treated within a universal healthcare system, with high-fidelity EIS provided during the critical period. This makes the identified trajectories and predictors more likely to be representative of real-world clinical populations and practices, potentially generalizable to other settings.

Future research should conduct external validation of latent trajectory models to ensure the robustness and generalizability of the findings, and to improve our understanding of the underlying mechanisms driving trajectories. This involves investigating whether identified latent structures, distributions, and predictors can be replicated across independent samples and extended to diverse outcome dimensions, such as neurobiological markers and more holistic outcomes like remission and recovery. Concerns have been raised about the sustainability of EIS benefits over time^29,118^ and further research should specifically test the assumption that outcome trajectories established during the critical period are maintained in the long-term.

### Conclusion

While reducing DUP and minimizing delays in antipsychotic initiation are critical first steps, they alone are not sufficient to improve clinical and functional trajectories for all individuals experiencing FEP. Optimizing EIS likely depends on the delivery of personalized, multi-component interventions, including cognitive and psychosocial interventions, not only at the earliest time during the critical period but also flexibly in a manner adapted to each individual’s evolving needs and circumstances. Precision psychiatry is an emerging field, and there are currently no established guidelines on how and when to tailor specific EIS interventions based on individual clinical profiles.^118,119^ Our findings offer insights into clinically meaningful subgroups to inform prognosis and lay the groundwork for the development of individually tailored EIS. Notably, the absence of early improvements in negative symptoms, specifically secondary negative symptoms, may be determinant in identifying at-risk individuals following antipsychotic initiation. Given the limited availability of specific interventions for negative symptoms, clinicians must first identify actionable factors that cause or contribute to secondary negative symptoms and provide appropriate interventions based on the case formulation.^85,86^ Addressing negative symptoms effectively may play a critical role in predicting and improving clinical and functional trajectories, potentially more so than positive symptoms alone.

## Supplementary Material

sbaf045_suppl_Supplementary_Tables
