# Supplementary material for: Latent Trajectories of Positive, Negative Symptoms and Functioning in Early Intervention Services for First-Episode Psychosis: A 2-Year Follow-Up Study
Source: Schizophr Bull. 2025 May 4;51(5):1428–42. doi: 10.1093/schbul/sbaf045 (PMC12414566; doi:10.1093/schbul/sbaf045)
Supplement: sbaf045_suppl_Supplementary_Tables [file sbaf045_suppl_supplementary_tables.pdf]

Supplementary Table 1. Summary of SAPS models.

Model	Latent classes	Parameters	Polynomial	Random effect	Residuals	BIC	AIC	AICC	CAIC	Class 1	Class 2	Class 3	Class 4	Class 5	Class 6	APPA1	APPA2	APPA3	APPA4	APPA5	APPA6	aLMR pval	Entropy	Errors
**GCM**																								
	**1**	**14**	**cubic**	**intercept, linear, quadratic, cubic**	**free across time**	**23776**	**23713**	**23713**	**23790**	**679 (100%)**						**1.00**								
**GBTM**																								
	**2**	**10**	**cubic**	**0**	**fix across time & classes**	**23508**	**23463**	**23463**	**23518**	**477 (70%)**	**202 (30%)**					**0.92**	**0.88**					**0.000**	**0.70**	
	3	15				23458	23390	23391	23473	463 (68%)	180 (27%)	36 (5%)				0.90	0.85	0.79				0.723	0.72	
	4	20				23425	23334	23335	23445	38 (6%)	81 (12%)	407 (60%)	153 (23%)			0.80	0.80	0.84	0.70			0.008	0.66	
	5	25				23411	23298	23300	23436	22 (3%)	61 (9%)	179 (26%)	29 (4%)	388 (57%)		0.76	0.80	0.70	0.82	0.85		0.344	0.69	
	6	30				23420	23284	23287	23450	60 (9%)	26 (4%)	83 (12%)	393 (58%)	102 (15%)	15 (2%)	0.77	0.84	0.64	0.83	0.64	0.83	0.190	0.66	
**LCGA**																								
	**2**	11	**cubic**	**0**	free across classes	23398	23349	23349	23409	302 (44%)	377 (56%)					0.90	0.89					0.000	0.66	
		18			free across time	23461	23380	23381	23479	472 (70%)	207 (30%)					0.92	0.90					0.000	0.73	
		**27**			**free across time & classes**	**22645**	**22523**	**22526**	**22672**	**469 (69%)**	**210 (31%)**					**0.97**	**0.93**					**0.000**	**0.86**	
**GMM (class-invariant)**																								
	**2**	11	**cubic**	intercept	fix across time & classes	23478	23429	23429	23489	505 (74%)	174 (26%)					0.90	0.85					0.000	0.64	
		12			free across classes	23325	23271	23271	23337	258 (38%)	421 (62%)					0.87	0.83					0.000	0.51	
		19			free across time	23388	23302	23303	23407	44 (6%)	635 (94%)					0.92	0.95					0.000	0.79	
		28			free across time & classes	22535	22408	22411	22563	224 (33%)	455 (67%)					0.92	0.95					0.000	0.80	
		13		**intercept, linear**	fix across time & classes	23443	23384	23385	23456	168 (25%)	511 (75%)					0.87	0.90					0.046	0.65	
		14			free across classes	23277	23214	23215	23291	388 (57%)	291 (43%)					0.86	0.89					0.000	0.60	out of range estimates
		21			free across time	23365	23270	23271	23386	500 (74%)	179 (26%)					0.90	0.87					0.444	0.66	
		**30**			**free across time & classes**	**22443**	**22308**	**22311**	**22473**	**438 (65%)**	**241 (35%)**					**0.94**	**0.91**					**0.000**	**0.78**	
		16		intercept, linear, quadratic	fix across time & classes	23430	23357	23358	23446	62 (9%)	617 (91%)					0.90	0.93					0.012	0.72	out of range estimates
		17			free across classes	23255	23178	23179	23272	303 (45%)	376 (55%)					0.89	0.84					0.000	0.59	out of range estimates
		24			free across time	23316	23208	23210	23340	614 (90%)	65 (10%)					0.93	0.90					0.005	0.72	out of range estimates
		33			free across time & classes	22448	22299	22302	22481	236 (35%)	443 (65%)					0.92	0.94					0.000	0.78	
				intercept, linear, quadratic, cubic	free across classes																			fails to converge
					free across time & classes																			fails to converge
					fix across time & classes																			fails to converge
					free across time																			fails to converge
**GMM (class-variant)**																								
	**2**	12	**cubic**	intercept	fix across time & classes	23474	23420	23420	23486	430 (63%)	249 (37%)					0.86	0.85					0.000	0.55	
		13			free across classes	23325	23266	23266	23338	386 (57%)	293 (43%)					0.82	0.88					0.000	0.52	
		20			free across time	23390	23299	23301	23410	313 (46%)	366 (54%)					0.88	0.86					0.009	0.58	out of range estimates
		29			free across time & classes	22468	22337	22340	22497	477 (70%)	202 (30%)					0.96	0.92					0.000	0.82	
		16		**intercept, linear**	fix across time & classes	23374	23301	23302	23390	338 (50%)	341 (50%)					0.82	0.88					0.020	0.54	out of range estimates
		17			free across classes	23262	23185	23186	23279	350 (52%)	329 (48%)					0.90	0.83					0.000	0.58	out of range estimates
		24			free across time	23174	23065	23067	23198	277 (41%)	402 (59%)					0.82	0.92					0.000	0.63	out of range estimates
		**33**			**free across time & classes**	**22418**	**22269**	**22272**	**22451**	**460 (68%)**	**219 (32%)**					**0.96**	**0.91**					**0.000**	**0.81**	
		22		intercept, linear, quadratic	fix across time & classes	23262	23163	23165	23284	247 (36%)	432 (64%)					0.83	0.90					0.004	0.62	out of range estimates
		39			free across time & classes	22424	22248	22253	22463	466 (69%)	213 (31%)					0.96	0.92					0.000	0.81	
					free across classes																			fails to converge
					free across time																			fails to converge
				intercept, linear, quadratic, cubic	free across classes																			fails to converge
					free across time & classes																			fails to converge
					fix across time & classes																			fails to converge
					free across time																			fails to converge

Note: The parameters and fit indices of the optimal model specification are indicated in **bold**. AIC: Akaike information criterion, Akaike information corrected criterion, CAIC: Consistent Akaike information criterion, aLMR: Adjusted Lo-Mendell-Rubin likelihood ratio test, APPA: Average posterior probability of assignment, BIC: Baeysian information criterion, GBTM: Group-based trajectory model, GMM: Growth mixture model, LL: Loglikelihood, SAPS: Scale for the assessment of positive symptoms.

1. SAPS model selection

Given the number of time (i.e., 9), a GCM was estimated with a pre-set polynomial order assumed to be cubic.

1.1. Class enumeration

GBTMs were fitted with the number of class ranging from $K = 2$ to an expected maximum of $K = 6$, as proposed by prior research. Across all fit statistics (i.e., BIC, AIC, CAIC, AICC), GBTMs with $K \geq 2$ classes consistently outperformed the single-class GCM. The best BIC value was observed for $K = 5$ classes. The BLRT draws did not converge to a reliable solution and were therefore not reported. However, the aLMR-LRT indicated that the $K = 5$ classes model ($p = 0.334$) could be further reduced in favour of a $K = 4$ classes model. The later demonstrated relatively low discrimination of trajectories, with APPA and class count only marginally above cut-off values, respectively APPA4 = 0.705 and class 1 = 38 (5.5%). While the $K = 4$ classes model could not be further reduced ($p = 0.008$), the K = 3 classes model was dismissed in favor of the $K = 2$ classes model ($p < 0.001$) which was therefore selected considering parsimony, better APPAs (> 0.88) and clinically interpretable class counts (> 5%).

1.2. Covariance structure

Given the $K = 2$ class structure, the best GBTM was extended into LCGAs, allowing different residual variance error structures. The LCGA with unrestricted residual variance across time and classes yielded the best BIC value ($BIC = 22645$) and was retained as it could not be further reduced ($p < 0.001$).

1.3. Random effects

GMMs were estimated by incrementally adding class-invariant and -variant random effect variances. Models that failed to converge, or provided out of range estimates were not further investigated. GMMs with free residual variance across time & classes along with intercept and linear slope class-invariant and -variant effects demonstrated best BIC values (respectively, $BIC = 22443$ and $BIC = 22418$) with significant aLMR tests ($p < 0.001$). Out of the best LCGA, class-invariant and -variant GMMs, the later outperformed the $K = 2$ GBTM across all fit indices (i.e., BIC, AIC, CAIC, AICC), and was selected for further refinement.

1.4. Polynomial order

Wald tests revealed that cubic terms across both classes were significant ($p < 0.001$) resulting in the final specification of the latent trajectories for the SAPS.

1.5. Final model

The final model consisted of a 2-class cubic GMM with class-variant random effects for the intercept and linear slope, along with unrestricted residual variance across classes and over time. The APPAs for each class of the final model were *APPA1* = 0.96 and *APPA2* = 0.91, indicating that individuals were confidently assigned to their most likely latent classes. Additionally, the model exhibited overall high class separation, as indicated by *sE* = 0.81. Finally, the multivariate skewness and kurtosis test indicated a non-significant kurtosis (M = 115.7, SD = 6.28, p = 0.180) but marginally significant right-skewness (M = 15.45, SD = 2.66, p = 0.04).

Supplementary Table 2. Summary of SANS models.

Model	Latent classes	Parameters	Polynomial	Random effect	Residuals	BIC	AIC	AICC	CAIC	Class 1	Class 2	Class 3	Class 4	Class 5	Class 6	APPA1	APPA2	APPA3	APPA4	APPA5	APPA6	aLMR pval	Entropy	Errors
**GCM**																								
	**1**	**14**	**cubic**	**intercept, linear, quadratic, cubic**	**free across time**	**22604**	**22541**	**22541**	**22618**	**679 (100%)**						**1.00**								
**GBTM**																								
	2	10				23129	23084	23084	23139	265 (39%)	414 (61%)					0.94	0.94					0.000	0.79	
	**3**	**15**				**22812**	**22744**	**22745**	**22827**	**297 (44%)**	**259 (38%)**	**123 (18%)**				**0.86**	**0.90**	**0.89**				**0.004**	**0.75**	
	4	20	**cubic**	**0**	**fix across time & classes**	22720	22630	22631	22740	65 (10%)	231 (34%)	250 (37%)	133 (20%)			0.79	0.79	0.86	0.89			0.179	0.71	
	5	25				22605	22492	22494	22630	65 (10%)	162 (24%)	25 (4%)	229 (34%)	198 (29%)		0.79	0.84	0.91	0.85	0.78		0.001	0.74	
	6	30				22564	22428	22431	22594	64 (9%)	128 (19%)	183 (27%)	196 (29%)	82 (12%)	26 (4%)	0.80	0.76	0.73	0.84	0.81	0.87	0.126	0.71	
**LCGA**																								
		**17**			**free across classes**	**22798**	**22721**	**22722**	**22815**	**130 (19%)**	**224 (33%)**	**325 (48%)**				**0.89**	**0.90**	**0.87**				**0.002**	**0.76**	
	3	23	**cubic**	**0**	free across time	22819	22715	22716	22842	122 (18%)	291 (43%)	266 (39%)				0.90	0.86	0.91				0.003	0.76	
		41			free across time & classes	22881	22696	22701	22922	326 (48%)	209 (31%)	144 (21%)				0.87	0.90	0.89				0.055	0.76	
**GMM (class-invariant)**																								
		16			fix across time & classes	22454	22382	22382	22470	225 (33%)	272 (40%)	182 (27%)				0.73	0.68	0.74				0.457	0.43	
		18		Intercept	free across classes	22411	22329	22330	22429	297 (44%)	216 (32%)	166 (24%)				0.67	0.80	0.77				0.109	0.47	
		24			free across time	22447	22339	22341	22471	526 (77%)	58 (9%)	95 (14%)				0.80	0.80	0.84				0.133	0.57	
		42			free across time & classes	22466	22276	22282	22508	166 (24%)	149 (22%)	364 (54%)				0.80	0.80	0.71				0.161	0.48	
		18			fix across time & classes	22363	22281	22282	22381	441 (65%)	22 (3%)	216 (32%)				0.80	0.80	0.79				0.021	0.58	
	**3**	**20**		**intercept, linear**	**free across classes**	**22286**	**22195**	**22197**	**22306**	**93 (14%)**	**394 (58%)**	**192 (28%)**				**0.79**	**0.75**	**0.74**				**0.000**	**0.47**	
		26			free across time	22369	22251	22253	22395	161 (24%)	494 (73%)	24 (4%)				0.80	0.83	0.79				0.040	0.61	
		44	**cubic**		free across time & classes	22369	22170	22177	22413	176 (26%)	108 (16%)	395 (58%)				0.77	0.76	0.76				0.656	0.52	
		21			fix across time & classes	22342	22247	22249	22363	528 (78%)	121 (18%)	30 (4%)				0.84	0.80	0.80				0.008	0.63	
		23		intercept, linear, quadratic	free across classes	22262	22158	22160	22285	435 (64%)	114 (17%)	130 (19%)				0.79	0.78	0.76				0.192	0.52	
		29			free across time	22341	22210	22213	22370	34 (5%)	532 (78%)	113 (17%)				0.77	0.84	0.81				0.007	0.63	
		47			free across time & classes	22326	22114	22121	22373	90 (13%)	323 (48%)	266 (39%)				0.78	0.76	0.75				0.501	0.53	
				intercept, linear, quadratic, cubic	free across classes																			fails to converge
					free across time & classes																			fails to converge
					fix across time & classes																			fails to converge
					free across time																			fails to converge
**GMM (class-variant)**																								
		18			fix across time & classes	22451	22370	22371	22469	185 (27%)	230 (34%)	264 (39%)				0.75	0.72	0.68				0.197	0.43	
		20		intercept	free across classes	22408	22318	22319	22428	277 (41%)	213 (31%)	189 (28%)				0.67	0.80	0.75				0.117	0.47	
		26			free across time	22449	22331	22333	22475	99 (15%)	61 (9%)	519 (76%)				0.83	0.80	0.80				0.102	0.58	
		44			free across time & classes	22446	22247	22253	22490	177 (26%)	342 (50%)	160 (24%)				0.80	0.70	0.79				0.160	0.48	
		24			fix across time & classes	22378	22269	22271	22402	289 (43%)	368 (54%)	22 (3%)				0.81	0.77	0.81				0.003	0.58	
	**3**	**26**		**intercept, linear**	**free across classes**	**22292**	**22174**	**22176**	**22318**	**213 (31%)**	**372 (55%)**	**94 (14%)**				**0.75**	**0.75**	**0.80**				**0.000**	**0.48**	
		32			free across time	22390	22245	22248	22422	417 (61%)	240 (35%)	22 (3%)				0.77	0.80	0.82				0.008	0.57	
		50	**cubic**		free across time & classes	22378	22152	22161	22428	222 (33%)	249 (37%)	208 (31%)				0.74	0.73	0.73				0.529	0.46	
		33			fix across time & classes	22384	22235	22238	22417	209 (31%)	26 (4%)	444 (65%)				0.72	0.85	0.85				0.120	0.61	out of range estimates
		35		intercept, linear, quadratic	free across classes	22299	22141	22145	22334	146 (22%)	152 (22%)	381 (56%)				0.69	0.80	0.73				0.148	0.47	out of range estimates
		41			free across time	22380	22194	22200	22421	165 (24%)	23 (3%)	491 (72%)				0.71	0.82	0.87				0.022	0.65	out of range estimates
		59			free across time & classes	22358	22091	22102	22417	386 (57%)	108 (16%)	185 (27%)				0.74	0.82	0.71				0.552	0.47	out of range estimates
				intercept, linear, quadratic, cubic	free across classes																			fails to converge
					free across time & classes																			fails to converge
					fix across time & classes																			fails to converge
					free across time																			fails to converge

Note: The parameters and fit indices of the optimal model specification are indicated in **bold**. AIC: Akaike information criterion, Akaike information corrected criterion, CAIC: Consistent Akaike information criterion, aLMR: Adjusted Lo-Mendell-Rubin likelihood ratio test, APPA: Average posterior probability of assignment, BIC: Baeysian information criterion, GBTM: Group-based trajectory model, GMM: Growth mixture model, LL: Loglikelihood, SANS: Scale for the assessment of positive symptoms.

2. SANS model selection

Given the number of time (i.e., 9), a GCM was estimated with a pre-set polynomial order assumed to be cubic.

2.1. Class enumeration

GBTMs were fitted with the number of class ranging from $K = 2$ to an expected maximum of $K = 6$, as proposed by prior research. The BLRT draws did not converge to a reliable solution and were therefore not reported. The best BIC value was observed for $K = 6$ classes. However, the aLMR-LRT indicated that the $K = 6$ classes model ($p = 0.126$) could be further reduced in favour of a $K = 5$ classes model. The latter had one class only accounting for 25 individuals (3.7% of the sample) and was therefore not retained to preserve interpretability. Finally, the $K = 4$ classes model could be further reduced ($p = 0.179$), in favour of the $K = 3$ classes model ($BIC = 22818$, $p < 0.001$) that could not be further reduced, determining the optimal number of classes.

2.2. Covariance structure

Given the $K = 3$ class-structure, the best GBTM was extended through LCGAs, allowing different residual variance error structures. The LCGA with unrestricted residual variance across classes yielded the best BIC value ($BIC = 22798$) and was retained as it could not be further reduced ($p = 0.002$).

2.3. Random effects

GMMs were estimated by incrementally adding class-invariant and -variant random effect variances. Models failing to converge, or providing out of range estimates were not further investigated. GMMs with free residual variance across classes along with intercept and linear slope class-invariant and -variant random effects demonstrated best BIC values (respectively, $BIC = 22286$ and $BIC = 22292$) with significant aLMR tests ($p < 0.001$). Out of the best LCGA, class-invariant and -variant GMMs, the class-invariant GMM outperformed the $K = 3$ GBTM across all fit indices (i.e., BIC, AIC, CAIC, AICC), and was selected for further refinement.

2.4. Polynomial order

Nonsignificant higher order polynomial terms were dropped in one class per iteration. Wald tests indicated that the cubic term was significant in class 1 ($p = 0.015$) and class 2 ($p = 0.001$), while it proved non-significant in class 3 ($p = 0.262$). Following to discarding the cubic term for this class, the quadratic term remained non-significant ($p = 0.130$) in the second iteration. In the third and final iteration, the linear term was found to be significant ($p < 0.001$) and was therefore retained in class 3 along with a cubic term in class 1 and 2.

2.5. Final model

The final model consisted of a 3-class GMM with cubic and quadratic growth along with class-invariant random effects for the intercept and linear slope, as well as unrestricted residual variance across classes. The APPAs for the classes of the final model ranged from 0.74 to 0.79, indicating that individuals were assigned to their most likely latent classes with satisfying confidence. Additionally, the model exhibited overall low to medium class-separation, as indicated by $sE = 0.48$. Finally, the multivariate skewness and kurtosis test indicated a non-significant kurtosis ($M = 108.26$, $SD = 6.46$, $p = 0.911$) and non-significant skewness ($M = 10.12$, $SD = 2.54$, $p = 0.950$) indicating within-class normality.

.

Supplementary Table 3. Summary of SOFAS models.

Model	Latent classes	Parameters	Polynomial	Random effect	Residuals	BIC	AIC	AICC	CAIC	Class 1	Class 2	Class 3	Class 4	Class 5	Class 6	APPA1	APPA2	APPA3	APPA4	APPA5	APPA6	aLMR pval	Entropy	Errors
**GCM**																								
	**1**	**8**	**quadratic**	**intercept, linear, quadratic**	**free across time**	**11232**	**11197**	**11197**	**11240**	**650 (100%)**						**1.00**								
**GBTM**																								
	**2**	**8**	quadratic	0	fix across time & classes	**11082**	**11046**	**11047**	**11090**	**280 (43%)**	**370 (57%)**					**0.81**	**0.84**					**0.000**	**0.51**	
	3	12				11075	11021	11022	11087	261 (40%)	339 (52%)	50 (8%)				0.82	0.78	0.74				0.238	0.61	
	4	16				11076	11004	11005	11092	225 (35%)	233 (36%)	71 (11%)	121 (19%)			0.69	0.70	0.75	0.67			0.015	0.51	
	5	20				11080	10991	10992	11100	5 (1%)	210 (32%)	225 (35%)	139 (21%)	71 (11%)		0.90	0.66	0.69	0.68	0.67		0.298	0.55	
	6	24				11088	10981	10983	11112	46 (7%)	207 (32%)	7 (1%)	222 (34%)	92 (14%)	76 (12%)	0.58	0.65	0.79	0.63	0.62	0.69	0.433	0.52	
**LCGA**																								
	2	**9**	quadratic	0	**free across classes**	**11083**	**11043**	**11043**	**11092**	**417 (64%)**	**233 (36%)**					**0.86**	**0.82**					**0.000**	**0.55**	
		10			free across time	11094	11050	11050	11104	363 (56%)	287 (44%)					0.84	0.81					0.000	0.51	
		13			free across time & classes	11069	11011	11012	11082	368 (57%)	282 (43%)					0.84	0.87					0.150	0.54	
**GMM (class-invariant)**																								
	2	**9**	quadratic	**intercept**	**fix across time & classes**	**11083**	**11042**	**11043**	**11092**	**256 (39%)**	**394 (61%)**					**0.81**	**0.81**					**0.000**	**0.47**	
		10			free across classes	11087	11042	11042	11097	414 (64%)	236 (36%)					0.84	0.81					0.001	0.52	
		11			free across time	11094	11045	11045	11105	257 (40%)	393 (60%)					0.82	0.81					0.000	0.48	
		14			free across time & classes	11074	11011	11012	11088	384 (59%)	266 (41%)					0.85	0.84					0.109	0.52	
		11		intercept, linear	fix across time & classes	11074	11025	11026	11085	82 (13%)	568 (87%)					0.87	0.94					0.000	0.75	out of range estimates
		12			free across classes	11078	11024	11025	11090	401 (62%)	249 (38%)					0.89	0.74					0.002	0.51	out of range estimates
		13			free across time	11063	11005	11005	11076	558 (86%)	92 (14%)					0.94	0.87					0.002	0.75	out of range estimates
		16			free across time & classes	10958	10887	10888	10974	126 (19%)	524 (81%)					0.84	0.98					0.000	0.83	out of range estimates
		15		intercept, linear, quadratic	free across classes	11072	11004	11005	11087	482 (74%)	168 (26%)					0.91	0.83					0.002	0.62	out of range estimates
		16			free across time	11082	11011	11012	11098	558 (86%)	92 (14%)					0.94	0.87					0.240	0.75	out of range estimates
		19			free across time & classes	10978	10893	10894	10997	126 (19%)	524 (81%)					0.84	0.98					0.000	0.83	out of range estimates
					fix across time & classes																			fails to converge
**GMM (class-variant)**																								
	2	10	quadratic	intercept	fix across time & classes	11083	11038	11039	11093	460 (71%)	190 (29%)					0.83	0.85					0.000	0.52	out of range estimates
		**11**			**free across classes**	**11083**	**11034**	**11034**	**11094**	**502 (77%)**	**148 (23%)**					**0.84**	**0.80**					**0.000**	**0.50**	
		12			free across time	11096	11042	11042	11108	204 (31%)	446 (69%)					0.82	0.84					0.001	0.52	out of range estimates
		15			free across time & classes	11080	11013	11014	11095	254 (39%)	396 (61%)					0.84	0.85					0.127	0.52	
		14		intercept, linear	fix across time & classes	11066	11003	11004	11080	342 (53%)	308 (47%)					0.81	0.88					0.001	0.51	out of range estimates
		15			free across classes	11070	11003	11004	11085	294 (45%)	356 (55%)					0.87	0.83					0.001	0.51	out of range estimates
		16			free across time	10953	10881	10882	10969	524 (81%)	126 (19%)					0.98	0.84					0.000	0.83	out of range estimates
		19			free across time & classes	10950	10865	10866	10969	524 (81%)	126 (19%)					0.98	0.84					0.000	0.83	out of range estimates
		22		intercept, linear, quadratic	free across time	10969	10871	10872	10991	126 (19%)	524 (81%)					0.84	0.98					0.173	0.83	out of range estimates
		25			free across time & classes	11119	11008	11010	11144	418 (64%)	232 (36%)					0.85	0.84					0.240	0.51	out of range estimates
					free across classes																			fails to converge
					fix across time & classes																			fails to converge

Note: The parameters and fit indices of the optimal model specification are indicated in **bold**. AIC: Akaike information criterion, Akaike information corrected criterion, CAIC: Consistent Akaike information criterion, aLMR: Adjusted Lo-Mendell-Rubin likelihood ratio test, APPA: Average posterior probability of assignment, BIC: Baeysian information criterion, GBTM: Group-based trajectory model, GMM: Growth mixture model, LL: Loglikelihood, SOFAS: Social occupational functioning scale.

3. SOFAS model selection

Model selection was conducted following the strategy presented by Van Der Nest et al., (2020). For reference, a growth curve model (GCM) was fitted before conducting class enumeration. Given the number of time points (i.e., 3), the pre-set polynomial order was assumed to be quadratic. GCMs are characterized by random effects for all growth factors, and unrestricted residual variances across time.

3.1. Class enumeration

Group-based trajectory models (GBTM) were fitted with class counts ranging from $K = 2$ to an expected maximum of $K = 6$, as proposed by prior research {Chang, 2018 #274212}. GBTMs are characterized by random effects set to zero, and residual variances constrained to be equal across time and classes. Across all fit statistics (i.e., BIC, AIC, CAIC, AICC), GBTMs with K ≥ 2 classes consistently outperformed the single-class GCM. The best BIC value was observed for K = 3 classes. Likelihood ratio tests (LRT) were then employed to determine whether that K-class model was dismissed in favour of a K-1 model. While bootstrap LRT (BLRT) draws did not converge to a reliable solution and were therefore not reported, the Lo-Mendell-Rubin adjusted LRT (aLMR-LRT) indicated that the $K = 3$ classes model ($p = 0.238$) could be further reduced in favour of an optimal $K = 2$ classes model that could not be further reduced ($p < 0.001$), determining the optimal number of classes.

3.2. Covariance structure

Given the $K = 2$ class structure, the best GBTM was extended through latent class growth analysis (LCGA), allowing different residual variance error structures (i.e. same over class but different across time, same over time but different over class, and different across time and over class). Although, the LCGA with unrestricted residual variance across time and classes yielded the best BIC value, the optimal class structure was rejected by the aLMR test ($p = 0.150$). One may consider that the decrease in BIC values as more parameters are added suggests a risk of model overfitting. Consequently, the LCGA model with the second-best BIC value ($BIC = 11083$), a significant aLMR test ($p < 0.001$), and unrestricted residual variance across classes was retained.

3.3. Random effects

Growth mixture models (GMM) were estimated by incrementally adding class-invariant and -variant random effect variances (i.e. first for the intercept, then for the intercept and linear slope, and finally for the intercept, linear and quadratic slope). However, as model complexity increased, more models failed to converge, or provided out of range estimates. Only GMMs featuring random intercept variance converged to a solution. The class-invariant GMM with fix residual variance across time & classes and, the class-variant GMM with free residual variance across classes demonstrated best BIC values (*BIC* = 11083) with significant aLMR tests ($p < 0.001$). Despite relaxing model constraints through LCGAs and class-invariant and -variant GMMs, none of these models outperformed the best fitting GBTM (*BIC* = 11082). Consequently, the GBTM with $K = 2$ classes was selected for further refinement.

3.4. Polynomial order

The significance of the higher polynomial term in the selected GBTM was confirmed for each class. Wald tests revealed that quadratic terms were significant across both classes ($p < 0.001$) leading to the final specification of the latent trajectories for the SOFAS.

3.5. Final model

The final model consisted of a 2-class quadratic GBTM with no random effects and with equal residual variance across classes and over time. The average posterior probabilities of assignment (APPA) for each class of the final model were *APPA1* = 0.81 and *APPA2* = 0.82, indicating that individuals were confidently assigned to their most likely latent classes. Additionally, the model exhibited overall satisfying class separation, as indicated by a scaled entropy (sE) of 0.51. Finally, the multivariate skewness and kurtosis test indicated a normal-range (i.e. ± 2) kurtosis ($M = 0.22$, $SD = 0.11$, $p < 0.001$) and non-significant skewness ($M = 14.67$, $SD = 0.53$, $p = 0.17$) indicating within-class normality.

Supplementary Table 4. Baseline characteristics across trajectories.

		SAPS			SANS				SOFAS	
Characteristic	N	**Fluctuating** (460; 68%)[1]	**Stable-low** (219; 32%)[1]	N	**Fluctuating** (104; 15%)[1]	**Decreasing** (277; 41%)[1]	**Stable-high** (298; 44%)[1]	N	**Stable-moderate** (280; 43%)[1]	**Increasing** (370; 57%)[1]
Age	679	23.2 (4.7)	24.7 (4.8)	679	23.2 (4.3)	23.5 (4.9)	24.0 (4.8)	650	23.5 (4.9)	23.7 (4.7)
Sex	679			679				650		
female		124 (27%)	81 (37%)		34 (33%)	94 (34%)	77 (26%)		68 (24%)	132 (36%)
male		336 (73%)	138 (63%)		70 (67%)	183 (66%)	221 (74%)		212 (76%)	238 (64%)
Ethnicity	632			632				601		
not visible minority		254 (60%)	137 (67%)		59 (61%)	158 (61%)	174 (63%)		157 (62%)	213 (61%)
visible minority		172 (40%)	69 (33%)		37 (39%)	101 (39%)	103 (37%)		96 (38%)	135 (39%)
IQ	488	96.3 (15.3)	98.8 (14.6)	488	97.5 (16.7)	98.9 (13.8)	95.3 (15.5)	472	95.0 (14.6)	98.7 (15.3)
Education (years)	413	12.2 (2.9)	13.1 (3.0)	413	12.3 (2.8)	12.9 (3.0)	12.3 (2.9)	398	11.9 (2.8)	13.0 (2.9)
Employment	625			625				594		
NEET		289 (69%)	124 (60%)		63 (64%)	169 (66%)	181 (67%)		204 (79%)	195 (58%)
non-NEET		130 (31%)	82 (40%)		35 (36%)	88 (34%)	89 (33%)		55 (21%)	140 (42%)
Housing	652			652				621		
dependent		210 (48%)	90 (43%)		48 (48%)	129 (48%)	123 (43%)		145 (55%)	152 (43%)
independent		231 (52%)	121 (57%)		52 (52%)	137 (52%)	163 (57%)		120 (45%)	204 (57%)
Relationship	670			670				639		
single		413 (91%)	186 (86%)		97 (95%)	243 (88%)	259 (88%)		254 (92%)	320 (88%)
not single		41 (9%)	30 (14%)		5 (5%)	32 (12%)	34 (12%)		22 (8%)	43 (12%)
SES	503	54.4 (14.6)	49.5 (16.4)	503	52.7 (13.8)	51.7 (15.5)	53.7 (15.8)	476	56.8 (13.1)	50.2 (16.2)
PAS	368	0.3 (0.1)	0.2 (0.1)	368	0.2 (0.1)	0.2 (0.1)	0.3 (0.1)	351	0.3 (0.1)	0.2 (0.1)
Age of onset	602	22.1 (4.7)	24.0 (4.7)	602	22.2 (3.9)	22.5 (5.0)	23.1 (4.8)	572	22.3 (4.5)	23.0 (5.0)
DUP (weeks) [2]	567	17.6 (120.9)	10.6 (84.3)	567	11.6 (129.0)	14.1 (93.4)	16.1 (117.1)	539	17.8 (120.3)	11.6 (88.6)
Mode of onset	587			587				558		
acute		78 (20%)	53 (26%)		23 (25%)	50 (21%)	58 (23%)		45 (20%)	78 (23%)
insidious		304 (80%)	152 (74%)		70 (75%)	191 (79%)	195 (77%)		179 (80%)	256 (77%)
Hospitalization	678			678				647		
outpatient on DOE		266 (58%)	108 (49%)		47 (46%)	163 (59%)	164 (55%)		124 (45%)	229 (62%)
inpatient on DOE		193 (42%)	111 (51%)		56 (54%)	114 (41%)	134 (45%)		154 (55%)	140 (38%)
Diagnosis	613			613				582		
affective psychosis		102 (25%)	78 (38%)		31 (32%)	77 (31%)	72 (27%)		62 (25%)	115 (34%)
SSD		305 (75%)	128 (62%)		66 (68%)	174 (69%)	193 (73%)		183 (75%)	222 (66%)
CPZeq	577	173.6 (163.6)	213.1 (217.3)	577	203.3 (155.3)	185.7 (173.4)	181.6 (202.7)	551	201.2 (182.2)	174.4 (183.9)
Adherence (%)	628	83.3 (33.8)	86.6 (30.6)	628	85.6 (31.8)	87.1 (29.5)	81.5 (35.8)	600	84.9 (31.9)	84.6 (33.0)
SOFAS	609	39.9 (12.8)	41.9 (13.2)	609	40.5 (13.8)	40.5 (12.8)	40.6 (12.9)	609	34.0 (9.4)	45.6 (13.0)
SAPS	673	11.5 (3.1)	11.1 (3.5)	673	11.5 (3.5)	11.7 (3.2)	11.1 (3.2)	642	12.0 (3.0)	11.1 (3.3)
SANS	674	10.1 (3.7)	9.6 (4.2)	674	9.8 (4.2)	10.5 (3.7)	9.4 (3.8)	643	11.3 (3.4)	9.0 (3.9)
HAS	523	9.5 (7.0)	8.8 (6.8)	523	8.6 (6.8)	9.6 (6.7)	9.2 (7.2)	494	10.1 (6.9)	8.8 (6.7)
CDS	652	5.1 (4.8)	5.0 (4.9)	652	4.4 (5.4)	5.6 (4.9)	4.8 (4.6)	622	5.1 (4.7)	5.0 (4.9)
SUMD	385	2.9 (1.2)	2.6 (1.2)	385	2.9 (1.3)	2.7 (1.2)	2.8 (1.2)	370	3.1 (1.2)	2.5 (1.2)
SUD	401			401				374		
no SUD		171 (63%)	84 (66%)		42 (64%)	105 (66%)	108 (61%)		91 (58%)	144 (66%)
past SUD		59 (22%)	31 (24%)		15 (23%)	39 (25%)	36 (20%)		35 (22%)	51 (23%)
current SUD		43 (16%)	13 (10%)		9 (14%)	15 (9%)	32 (18%)		30 (19%)	23 (11%)
Verbal memory	480	-1.2 (1.2)	-1.0 (1.1)	480	-1.0 (1.1)	-0.9 (1.2)	-1.4 (1.3)	464	-1.4 (1.2)	-0.9 (1.2)
Working memory	481	-0.8 (1.1)	-0.6 (0.9)	481	-0.7 (1.0)	-0.6 (0.9)	-0.8 (1.1)	465	-0.9 (1.2)	-0.6 (0.9)
Executive functioning	485	-1.2 (1.6)	-1.0 (1.4)	485	-1.1 (1.3)	-1.0 (1.4)	-1.4 (1.7)	469	-1.3 (1.5)	-1.0 (1.5)
Speed of processing	483	-0.4 (1.5)	-0.4 (1.1)	483	-0.5 (1.0)	-0.3 (1.0)	-0.5 (1.7)	467	-0.3 (1.7)	-0.4 (1.0)
Visual memory	465	-0.9 (1.2)	-0.8 (1.3)	465	-0.9 (1.3)	-0.9 (1.2)	-0.9 (1.3)	450	-0.9 (1.2)	-0.9 (1.3)
Visual attention	477	-0.5 (0.7)	-0.3 (0.8)	477	-0.4 (0.7)	-0.3 (0.7)	-0.5 (0.8)	461	-0.5 (0.7)	-0.4 (0.8)
Social cognition	431	-0.8 (1.3)	-0.7 (1.2)	431	-0.8 (1.2)	-0.6 (1.3)	-0.9 (1.3)	418	-0.9 (1.3)	-0.6 (1.3)
Early reponse (PS)	561			561				543		
no response		132 (36%)	7 (4%)		21 (23%)	40 (17%)	78 (33%)		70 (30%)	63 (20%)
early response		230 (64%)	192 (96%)		69 (77%)	195 (83%)	158 (67%)		162 (70%)	248 (80%)
Early reponse (NS)	558			558				540		
no response		300 (84%)	126 (63%)		57 (65%)	147 (63%)	222 (94%)		197 (85%)	215 (70%)
early response		59 (16%)	73 (37%)		31 (35%)	87 (37%)	14 (6%)		36 (15%)	92 (30%)
Early reponse (total)	559			559				541		
no response		228 (63%)	52 (26%)		38 (42%)	81 (35%)	161 (68%)		136 (59%)	133 (43%)
early response		132 (37%)	147 (74%)		52 (58%)	152 (65%)	75 (32%)		96 (41%)	176 (57%)

Note: [1]Mean (SD); n (%); [2]Median (SD)

CDS: Calgary depression scale, CPZeq: Chlorpromazine equivalent, DUP: Duration of untreated psychosis, HAS: Hamilton anxiety scale, NEET: Not in employment, education or training, NS: Negative symptoms, PAS: Premorbid adjustment scale, PS: Positive symptoms, SANS: Scale for the assessment of negative symptoms, SAPS: Scale for the assessment of positive symptoms, SES: Socioeconomic status, SOFAS: Social occupational functioning scale, SUD: Substance use disorder, SSD: Schizophrenia spectrum disorder, SUMD: Scale to assess awareness in mental disorder, YMRS: Young mania rating scale.

Supplementary Table 5. Non-Significant Baseline Predictors After Bonferroni Correction

Baseline predictor	Estimated mean			OR [95% CI]	p	
**SAPS**	*Fluctuating*	*Stable-low*				
Sex (male)	0.74	0.62		0.59 [0.4, 0.87]	0.008	**
Ethnicity (visible minority)	0.41	0.33		0.71 [0.47, 1.06]	0.095	
Education (years)	12.20	13.14		1.11 [1.03, 1.2]	0.01	*
Employment (non-NEET)	0.30	0.41		1.55 [1.04, 2.31]	0.031	*
Housing (independent)	0.52	0.58		1.26 [0.86, 1.84]	0.235	
Relationship (not single)	0.09	0.15		1.74 [0.98, 3.08]	0.058	
Negative symptoms	10.08	9.54		0.96 [0.92, 1.01]	0.161	
Anxiety symptoms	9.53	8.72		0.98 [0.95, 1.01]	0.272	
Depressive symptoms	5.13	4.96		0.99 [0.95, 1.06]	0.718	
Unawareness	2.91	2.53		0.76 [0.62, 0.94]	0.01	*
Mode of onset (insidious)	0.80	0.74		0.7 [0.45, 1.11]	0.13	
Substance use disorder	0.54	0.44		0.82 [0.59, 1.15]	0.259	
Hospitalization	0.42	0.52		1.49 [1.03, 2.16]	0.035	*
Adherence (%)	83.16	86.93		1 [1, 1.01]	0.247	
Chlorpromazine equivalent	172.00	216.67		1 [1, 1]	0.015	*
Premorbid adjustment	0.25	0.22		0.19 [0.03, 1.24]	0.084	
Functioning	39.81	42.14		1.01 [1, 1.03]	0.067	
IQ	96.15	99.02		1.01 [1, 1.03]	0.076	
Speed of processing	-0.41	-0.45		0.98 [0.85, 1.13]	0.766	
Visual attention	-0.49	-0.28		1.44 [1.07, 1.95]	0.017	*
Visual memory	-0.93	-0.84		1.06 [0.88, 1.28]	0.524	
Verbal memory	-1.24	-0.95		1.21 [1.01, 1.45]	0.034	*
Working memory	-0.80	-0.56		1.27 [1.01, 1.59]	0.036	*
Executive functioning	-1.24	-1.00		1.12 [0.97, 1.29]	0.134	
Social cognition	-0.80	-0.68		1.07 [0.9, 1.28]	0.439	
**SANS**	*Fluctuating*	*Deacreasing*	*Stable-high*			
Sex (male)	0.66	0.64	0.78			
Decreasing				0.49 [0.25, 0.96]	0.039	*
Fluctuating				0.56 [0.25, 1.24]	0.152	
Age	23.09	23.33	24.28			
Fluctuating				0.95 [0.89, 1.01]	0.12	
Decreasing				0.96 [0.9, 1.02]	0.221	
Ethnicity (visible minority)	0.39	0.40	0.36			
Fluctuating				1.12 [0.53, 2.36]	0.769	
Decreasing				1.15 [0.61, 2.15]	0.663	
Education (years)	12.20	13.10	12.15			
Fluctuating				1 [0.86, 1.17]	0.97	
Decreasing				1.12 [0.98, 1.27]	0.106	
Employment (non-NEET)	0.36	0.34	0.32			
Decreasing				1.11 [0.58, 2.12]	0.764	
Fluctuating				1.22 [0.57, 2.59]	0.613	
Housing (independent)	0.51	0.50	0.60			
Decreasing				0.67 [0.36, 1.22]	0.193	
Fluctuating				0.71 [0.35, 1.45]	0.348	
Relationship (not sinlge)	0.03	0.13	0.13			
Fluctuating				0.19 [0.02, 1.77]	0.146	
Decreasing				0.99 [0.42, 2.3]	0.972	
Socioeconomic status	52.60	50.92	54.71			
Decreasing				0.98 [0.96, 1.01]	0.195	
Fluctuating				0.99 [0.96, 1.02]	0.489	
Positive symptoms	11.52	11.94	10.81			
Fluctuating				1.07 [0.95, 1.2]	0.255	
Decreasing				1.12 [1.02, 1.22]	0.019	*
Depressive symptoms	4.06	6.00	4.72			
Fluctuating				0.97 [0.86, 1.09]	0.591	
Decreasing				1.05 [0.99, 1.12]	0.079	
Anxiety symptoms	8.35	9.84	9.22			
Fluctuating				0.98 [0.92, 1.04]	0.552	
Decreasing				1.01 [0.96, 1.06]	0.614	
Unawareness	2.99	2.58	2.86			
Decreasing				0.82 [0.59, 1.14]	0.235	
Fluctuating				1.09 [0.75, 1.6]	0.654	
Duration of untreated psychosis	2.24	2.60	2.83			
Fluctuating				0.83 [0.66, 1.04]	0.11	
Decreasing				0.93 [0.78, 1.1]	0.384	
Onset	22.05	22.30	23.48			
Fluctuating				0.94 [0.88, 1]	0.053	
Decreasing				0.95 [0.88, 1.02]	0.166	
Mode of onset (insidious)	0.74	0.80	0.77			

Fluctuating				0.87 [0.37, 2.06]	0.759	
Decreasing				1.26 [0.58, 2.74]	0.568	
Hospitalization	1.58	1.38	1.45			
Decreasing				0.77 [0.42, 1.4]	0.384	
Fluctuating				1.73 [0.85, 3.53]	0.13	
Schizophrenia-spectrum disorder	0.67	0.69	0.75			
Fluctuating				0.68 [0.31, 1.52]	0.352	
Decreasing				0.74 [0.37, 1.48]	0.392	
Substance use disorder	0.50	0.39	0.62			
Decreasing				0.64 [0.38, 1.08]	0.094	
Fluctuating				0.8 [0.44, 1.46]	0.465	
Chlorpromazine equivalent	215.89	186.73	170.06			
Fluctuating				1 [1, 1]	0.518	
Decreasing				1 [1, 1]	0.733	
Adherence (%)	86.15	88.87	79.02			
Fluctuating				1.01 [0.99, 1.02]	0.269	
Decreasing				1.01 [1, 1.02]	0.055	
Early response (PS)	0.77	0.89	0.60			
Fluctuating				2.27 [0.96, 5.33]	0.061	
Premorbid adjustment	0.23	0.21	0.27			
Decreasing				0.03 [0, 0.46]	0.012	*
Fluctuating				0.09 [0, 10.97]	0.325	
Functioning	40.50	40.50	40.65			
Fluctuating				1 [0.97, 1.03]	0.963	
Decreasing				1 [0.98, 1.02]	0.948	
IQ	97.82	99.93	93.90			
Fluctuating				1.02 [0.99, 1.05]	0.279	
Decreasing				1.03 [1, 1.05]	0.018	*
Speed of processing	-0.55	-0.13	-0.64			
Fluctuating				1.1 [0.74, 1.63]	0.636	
Decreasing				1.52 [0.99, 2.32]	0.057	
Visual attention	-0.43	-0.22	-0.61			
Fluctuating				1.37 [0.74, 2.51]	0.315	
Decreasing				2.03 [1, 4.09]	0.049	*
Visual memory	-0.93	-0.84	-0.94			
Fluctuating				1.01 [0.72, 1.41]	0.973	
Decreasing				1.06 [0.8, 1.41]	0.678	
Verbal memory	-0.95	-0.75	-1.64			
Fluctuating				1.63 [1.13, 2.36]	0.009	**
Working memory	-0.69	-0.58	-0.88			
Fluctuating				1.19 [0.83, 1.7]	0.357	
Decreasing				1.33 [0.94, 1.88]	0.109	
Executive functioning	-1.09	-0.80	-1.56			
Fluctuating				1.22 [0.93, 1.59]	0.146	
Decreasing				1.42 [1.06, 1.92]	0.02	*
Social cognition	-0.84	-0.53	-0.95			
Fluctuating				1.07 [0.78, 1.46]	0.688	
Decreasing				1.29 [0.95, 1.75]	0.101	
**SOFAS**	*Stable-moderate*	*Increasing*				
Sex	1.79	1.62		0.43 [0.25, 0.75]	0.003	**
Age	23.52	23.76		1.01 [0.96, 1.06]	0.682	
Ethnicity (Visible minority)	0.37	0.39		1.05 [0.63, 1.75]	0.838	
Education (years)	11.49	13.28		1.26 [1.09, 1.47]	0.003	**
Housing (independent)	0.44	0.62		2.09 [1.27, 3.44]	0.004	**
Relationship (not single)	0.07	0.13		1.99 [0.83, 4.78]	0.126	
Depressive symptoms	5.16	5.01		0.99 [0.95, 1.04]	0.79	
Anxiety symptoms	10.40	8.38		0.96 [0.92, 1]	0.043	*
Duration of untreated psychosis	3.00	2.31		0.8 [0.68, 0.94]	0.006	**
Onset	22.06	23.19		1.05 [0.99, 1.11]	0.08	
Mode of onset (insidious)	1.80	1.76		0.75 [0.4, 1.4]	0.363	
Schizophrenia-spectrum disorder	0.78	0.65		0.52 [0.29, 0.92]	0.024	*
Substance use disorder	0.65	0.40		0.63 [0.41, 0.97]	0.038	*
Chlorpromazine equivalent	214.73	164.82		1 [1, 1]	0.133	
Adherence (%)	84.66	84.18		1 [0.99, 1.01]	0.902	
Early response (PS)	0.67	0.82		2.25 [1.21, 4.19]	0.01	*
IQ	94.05	99.54		1.03 [1.01, 1.05]	0.009	**
Speed of processing	-0.35	-0.48		0.93 [0.79, 1.09]	0.348	
Visual attention	-0.50	-0.35		1.3 [0.9, 1.89]	0.162	
Visual memory	-0.95	-0.86		1.06 [0.84, 1.33]	0.635	
Working memory	-0.96	-0.54		1.51 [1.1, 2.06]	0.01	*
Executive functioning	-1.45	-0.93		1.26 [1.03, 1.55]	0.025	*
Social cognition	-1.00	-0.56		1.31 [1.02, 1.68]	0.037	*

Supplementary Table 6. Distal characteristics across trajectories.

	SAPS			SANS				SOFAS		
**Characteristic**	**N**	**Fluctuating** (460; 68%)[1]	**Stable-low** (219; 32%)[1]	**N**	**Fluctuating** (104; 15%)[1]	**Decreasing** (277; 41%)[1]	**Stable-high** (298; 44%)[1]	**N**	**Stable-moderate** (280; 43%)[1]	**Increasing** (370; 57%)[1]
CPZeq	450	260.1 (285.8)	104.6 (126.6)	450	208.5 (225.6)	159.4 (182.3)	248.7 (308.3)	435	276.7 (293.1)	155.5 (207.6)
Adherence	360	81.5 (34.6)	81.4 (35.4)	360	77.1 (36.9)	78.7 (36.7)	85.9 (31.6)	349	83.6 (32.7)	80.3 (35.8)
SOFAS	294	57.2 (16.7)	74.8 (14.8)	294	64.5 (15.1)	68.1 (18.3)	58.8 (18.0)	294	49.2 (12.5)	77.2 (10.6)
SAPS	404	4.7 (4.2)	0.8 (1.1)	404	3.3 (4.2)	3.1 (3.8)	3.5 (3.9)	395	4.5 (4.0)	2.4 (3.5)
SANS	404	7.1 (4.3)	3.7 (3.5)	404	6.0 (4.2)	5.0 (3.8)	6.8 (4.6)	394	8.4 (4.2)	4.0 (3.3)
HAS	347	3.4 (4.6)	2.0 (3.5)	347	3.5 (4.4)	2.9 (4.1)	2.8 (4.4)	338	3.4 (4.5)	2.6 (4.1)
CDS	352	1.6 (2.7)	0.6 (1.6)	352	2.1 (3.5)	0.8 (1.8)	1.3 (2.4)	343	1.7 (2.8)	0.9 (1.9)
SUMD	196	2.3 (1.2)	1.9 (1.1)	196	2.2 (1.2)	2.1 (1.2)	2.2 (1.1)	191	2.5 (1.3)	2.0 (1.0)
Remission of positive symptoms	396			396				386		
no remission		170 (64%)	6 (5%)		30 (48%)	56 (35%)	90 (52%)		105 (60%)	66 (31%)
remission		96 (36%)	124 (95%)		32 (52%)	106 (65%)	82 (48%)		69 (40%)	146 (69%)
Remission of negative symptoms	412			412				400		
no remission		221 (80%)	65 (47%)		55 (83%)	95 (57%)	136 (76%)		164 (90%)	114 (52%)
remission		54 (20%)	72 (53%)		11 (17%)	71 (43%)	44 (24%)		18 (10%)	104 (48%)
Clinical remission	423			423				411		
no remission		242 (85%)	68 (50%)		59 (86%)	104 (62%)	147 (79%)		177 (94%)	125 (56%)
remission		44 (15%)	69 (50%)		10 (14%)	65 (38%)	38 (21%)		12 (6%)	97 (44%)
Functional remission	351			351				352		
no remission		215 (87%)	41 (39%)		46 (82%)	72 (55%)	138 (84%)		209 (100%)	48 (34%)
remission		32 (13%)	63 (61%)		10 (18%)	59 (45%)	26 (16%)		0 (0%)	95 (66%)
Recovery	434			434				427		
no recovery		293 (95%)	81 (65%)		69 (95%)	122 (75%)	183 (92%)		220 (100%)	147 (71%)
recovery		17 (5%)	43 (35%)		4 (5%)	41 (25%)	15 (8%)		0 (0%)	60 (29%)

[1]Mean (SD); n (%)

CDS: Calgary depression scale, CPZeq: Chlorpromazine equivalent, DUP: Duration of untreated psychosis, HAS: Hamilton anxiety scale, NEET: Not in employment, education or training, NS: Negative symptoms, PAS: Premorbid adjustment scale, PS: Positive symptoms, SANS: Scale for the assessment of negative symptoms, SAPS: Scale for the assessment of positive symptoms, SES: Socioeconomic status, SOFAS: Social occupational functioning scale, SUD: Substance use disorder, SSD: Schizophrenia spectrum disorder, SUMD: Scale to assess awareness in mental disorder, YMRS: Young mania rating scale.

Supplementary Table 7. Sensitivity analysis - Summary of best-fitting latent growth mixture models.

Outcome	N	Latent classes	Model	Parameters	Polynomial	Random effect	Residuals	BIC	aLMR pval	Class 1	Class 2	Class 3	Entropy
SOFAS	460	2	GBTM	8	quadratic	0	fix across time & classes	9527	< 0.001	197 (43%)	263 (57%)		0.66
SAPS	458	2	GMM (class-variant)	33	cubic	intercept & linear slope	free across time & classes	18708	< 0.001	295 (64%)	163 (36%)		0.90
SANS	456	3	GMM (class-invariant)	18	cubic (1, 2); linear (3)	intercept & linear slope	free across classes	18500	< 0.001	66 (14%)	182 (40%)	208 (46%)	0.53

Note: aLMR: Adjusted Lo-Mendell-Rubin likelihood ratio test, BIC: Baeysian information criterion, GBTM: Group-based trajectory model, GMM: Growth mixture model, SANS: Scale for the assessment of negative symptoms, SAPS: Scale for the assessment of positive symptoms, SOFAS: Social occupational functioning scale.
